# Kidney cancer biomarkers and targets for therapeutics: survivin (BIRC5), XIAP, MCL-1, HIF1α, HIF2α, NRF2, MDM2, MDM4, p53, KRAS and AKT in renal cell carcinoma

**DOI:** 10.1186/s13046-021-02026-1

**Published:** 2021-08-12

**Authors:** Fengzhi Li, Ieman A. M. Aljahdali, Renyuan Zhang, Kent L. Nastiuk, John J. Krolewski, Xiang Ling

**Affiliations:** 1 Department of Pharmacology & Therapeutics, Roswell Park Comprehensive Cancer Center, Buffalo, New York 14263 USA; 2 Genitourinary Disease Site Research Group, Roswell Park Comprehensive Cancer Center, Buffalo, New York 14263 USA; 3 Kidney Cancer Research Interest Group, Roswell Park Comprehensive Cancer Center, Buffalo, New York 14263 USA; 4 Developmental Therapeutics (DT) Program, Roswell Park Comprehensive Cancer Center, Buffalo, New York 14263 USA; 5 Department of Cellular & Molecular Biology, Roswell Park Comprehensive Cancer Center, Buffalo, New York 14263 USA; 6 Department of Cancer Genetics & Genomics, Roswell Park Comprehensive Cancer Center, Buffalo, New York 14263 USA; 7 Department of Urology, Roswell Park Comprehensive Cancer Center, Buffalo, New York 14263 USA; 8 Canget BioTekpharma LLC, Buffalo, New York 14203 USA

**Keywords:** Renal cell carcinoma (RCC), Survivin (BIRC5), Hypoxia inducible factor (HIF), Nuclear factor erythroid 2-related factor 2 (NRF2), TP53/p53, AKT, MCL-1, XIAP, MDM2, MDM4

## Abstract

The incidence of renal cell carcinoma (RCC) is increasing worldwide with an approximate 20% mortality rate. The challenge in RCC is the therapy-resistance. Cancer resistance to treatment employs multiple mechanisms due to cancer heterogeneity with multiple genetic and epigenetic alterations. These changes include aberrant overexpression of (1) anticancer cell death proteins (e.g., survivin/BIRC5), (2) DNA repair regulators (e.g., ERCC6) and (3) efflux pump proteins (e.g., ABCG2/BCRP); mutations and/or deregulation of key (4) oncogenes (e.g., MDM2, KRAS) and/or (5) tumor suppressor genes (e.g., TP5/p53); and (6) deregulation of redox-sensitive regulators (e.g., HIF, NRF2). Foci of tumor cells that have these genetic alterations and/or deregulation possess survival advantages and are selected for survival during treatment. We will review the significance of survivin (BIRC5), XIAP, MCL-1, HIF1α, HIF2α, NRF2, MDM2, MDM4, TP5/p53, KRAS and AKT in treatment resistance as the potential therapeutic biomarkers and/or targets in RCC in parallel with our analized RCC-relevant TCGA genetic results from each of these gene/protein molecules. We then present our data to show the anticancer drug FL118 modulation of these protein targets and RCC cell/tumor growth. Finally, we include additional data to show a promising FL118 analogue (FL496) for treating the specialized type 2 papillary RCC.

**Supplementary Information:**

The online version contains supplementary material available at 10.1186/s13046-021-02026-1.

## Background

Renal cell carcinoma (RCC) can be roughly classified into 3 major histological subtypes: clear-cell RCC (ccRCC 70–80%), papillary RCC (pRCC, 10–15%) [1], and chromophobe RCC (chRCC, 5–10%) [2] plus rare subtypes of collecting duct RCC (cdRCC) and sarcomatoid RCC (srRCC) [3]. The major driver for ccRCC is the genetic or epigenetic loss of VHL, which results in the dysregulation of hypoxia inducible factor (HIF) signaling. Most type 1 pRCC is associated with met oncogene activity, whereas type 2 pRCC is diverse with a variety of genetic and epigenetic perturbations, including CNKN2A silencing, SETD2 mutations, and TFE fusions. For chRCC, it typically has abundant mitochondria, mitochondrial DNA mutations, and metabolic reprogramming but few dominant somatic mutations along with frequent loss of a single copy of multiple chromosomes. For cdRCC and srRCC, they have similar genomics to the type 2 pRCC, with the addition of mutations to SETD2 and SMARCB1 in up to half of the cdRCC tumors examined, and BAP1, PTEN, and NF2 in the sarcomatoid tumors.

With the above RCC general information provided, we believe that the goal of basic and translational cancer research is to develop effective and targeted cancer therapies. RCC has a high mortality rate with increasing incidence over time. Specifically, the estimated incidence continuously increased each year for RCC, especially in the last 3 years (2019–2021, Table 1). Such an increase is projected to continue its advance to over 50% in the next 20 years [16]. Intriguingly, the estimated death rate resulting from RCC in the United States has had a significant percentage decrease over the past 3 years, especially for the current Year 2021 (Table 1), and is projected to continue to decline going forward [16]. This promising estimation of the decrease in RCC patients’ death rates in the US is likely attributed to the active development of new treatment approaches and early diagnostic methods from preclinical and clinical studies in the past 5 years or so. For example, immunotherapies using immune checkpoint inhibitors (ICIs), therapeutic vaccines and adoptive cell therapies are currently active research areas. ICIs have been approved for RCC [17], and new agents are being tested in preclinical studies and clinical trials for the treatment of RCC [18]. The progressing development of ICIs in preclinical studies and clinical trials [19] as well as the practical knowledge and understanding of immunotherapies have significantly improved [20], and give us new hope. Additionally, growing research in the area of cancer metabolism for an effort to identify oncometabolites for early cancer diagnosis and prognosis gives additional hope to cancer patients, especially for RCC patients [21]. For example, changes in oncometabolites (e.g., fumarate, succinate and D/L-2-hydroxyglutarate) could potentially be exploited for the development of novel biomarkers and targets for RCC patients [21].

**Table 1:** The yearly RCC patients’ incidence and death estimation in USA

Year	Total RCC cases	Total death	Death rate (%)	References
2021	76,080	13,780	18.1	[4]
2020	73,750	14,830	20.1	[5]
2019	73,820	14,770	20.0	[6]
2018	65,340	14,970	22.9	[7]
2017	63,990	14,400	22.5	[8]
2016	62,700	14,240	22.7	[9]
2015	61,560	14,080	22.9	[10]
2014	63,920	13,860	21.7	[11]
2013	65,150	13,680	21.0	[12]
2012	64,770	13,570	20.8	[13]
2011	60,920	13,120	21.5	[14]
2010	58,240	13,040	22.4	[15]

Nevertheless, it appears that with regard to using immunotherapy, only a small percentage of RCC patients might benefit from such treatments in most cases [20]. In fact, most RCC patients treated with immunotherapy failed to achieve a durable benefit and finally the cancer acquired resistance to treatment [20]. Additionally, the cancer metabolism-based treatment and diagnosis are still in early stages and require further development for possible clinical application [21]. Furthermore, many RCC patients have already developed into advanced and metastatic disease at the time of diagnosis. For these and additional reasons, a significant percentage of RCC patients would not receive valuable benefits from these new therapies. Therefore, additional novel treatment paradigms and therapeutic strategies are still needed for the treatment of RCC patients, especially for those with advanced and metastatic diseases.

In this article, based on our knowledge we have selected a set of unique biomarkers and targets that are potentially relevant to RCC but have not been comparatively reviewed in RCC. In other words, we have reviewed a set of genes and/or their proteins as potential RCC biomarkers and therapeutic targets together with our analyzed TCGA data and innovative data from our research group that are relevant to RCC. It is our hope that this article may suggest potential new strategies, combinational regimens, and/or conceptual paradigms and may lead to possible research extensions out of the previous box or the development of alternative solutions leading to novel therapeutics for RCC patients.

## Survivin (BIRC5)

### Survivin as a biomarker for RCC prediction and prognosis

Early studies on the expression of survivin (also called BIRC5) indicated that survivin expression is an independent predictor of clear cell RCC (ccRCC) progression and death and may provide a novel target for the development of new adjuvant therapies [22]. Further studies have indicated that the expression of survivin together with the expression of B7-H1, a ubiquitous antiapoptotic receptor, provides an even better prediction of ccRCC tumor aggressiveness [23]. For example, the studies found that there were 177 (59.4%) survivin (Low)/B7-H1(−), 51 (17.1%) survivin (Hi)/B7-H1(−), 29 (9.7%) survivin (Low)/B7-H1(+), and 41 (13.8%) survivin (Hi)/B7-H1(+) tumors [23]. The 5-year cancer-specific survival rates for the ccRCC patients within each group were 89.3, 59.7, 70.0, and 16.2%, respectively [23]. The prognostic and clinicopathological significance of survivin expression for renal cancer patients’ outcomes were further validated through systematic review and meta-analysis of the early studies on survivin expression in renal cancer [24–26]. Interestingly, Parker et al. used immunohistochemistry (IHC) to determine the expression level of B7-H1, survivin, and Ki-67 for 634 consecutive ccRCC patients and then combined the 3 independent predictors of ccRCC outcomes into a single scoring panel termed as “BioScore” with the hopes to refine outcome prediction [27]. However, using the BioScore prognostic algorithms, Hutterer et al. studied a cohort of 393 nonmetastatic RCC patients and found that although a higher BioScore was significantly associated with a higher cancer-specific mortality, the magnitude of this association was weak and not independent from other prognosticators used [28]. Therefore, these authors concluded that BioScore did not improve the prognostic accuracy of the Mayo Clinic stage, size, grade and necrosis score [28]. Nevertheless, using The Cancer Genome Atlas (**TCGA**) database for identifying autophagy-related genes (ARGs), a recent study revealed that the two key ARGs, CASP4 and BIRC5/survivin are independently and negatively associated with renal cancer patients’ survival [29].

**Fig. 1:**
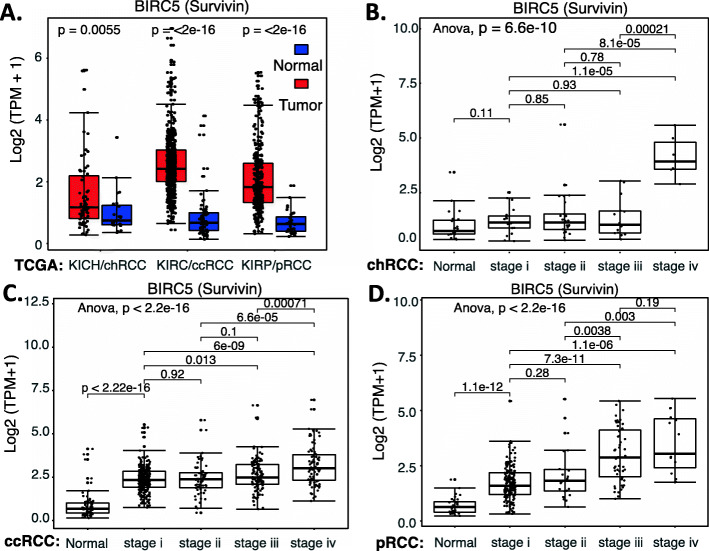
Survivin expression in RCC tumor tissues versus in the associated normal tissues: Boxplots of the BIRC5 (survivin) expression level across TCGA-RCC subtypes in renal tumor (red) versus the associated normal renal tissues (blue) were presented (**A**). BIRC5/survivin expression among different stages of chRCC (**B**), ccRCC (**C**) and pRCC (**D**) versus associated normal tissues was box-plotted. BIRC5/survivin expression was presented in the log2 (TPM + 1) scale format. Data were presented as the mean ± standard deviation (SD). A t-test was used to evaluate the statistical significance of the mRNA expression level in normal renal versus tumor tissues. One-way ANOVA was used to compare BIRC5/survivin expression among normal renal tissues versus different stages of RCC tumor tissues. The figure was performed using R version 4.0.3

**Fig. 2:**
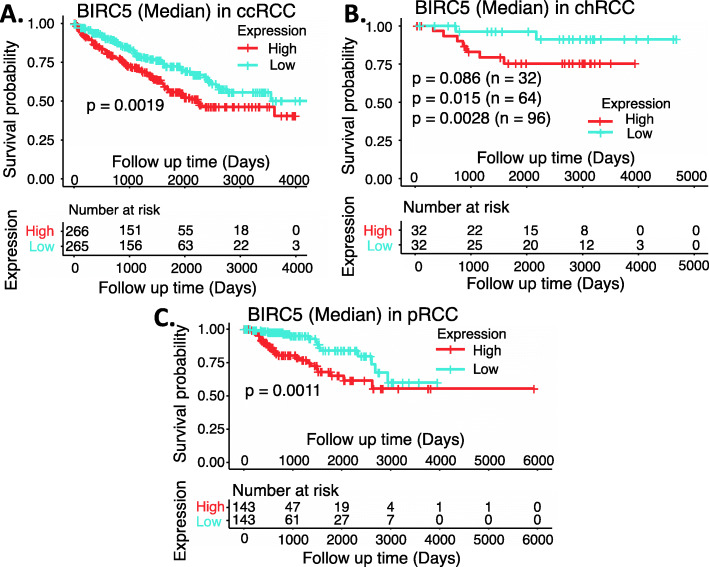
Effects of survivin/BIRC expression on RCC patient survival probability: Kaplan-Meier survival analyses of overall survival (OS) from TCGA-RCC cohorts of ccRCC (**A**), chRCC (**B**) and pRCC (**C**) were presented. Patients were grouped into the high survivin expression group versus the low survivin expression group based on the median mRNA expression of the BIRC5/survivin gene. Each *p*-value for the significance from high versus low BIRC5/survivin gene expression was calculated using the log-rank test. The figures were performed using R version 4.0.3

In order to better understand the survivin prognostic biomarker role in RCC, we have downloaded the publicly available kidney tumor versus normal sample datasets from UCSC Xena browser. This includes the cohorts of (1) GDC TCGA Kidney Chromophobe/KICH (chRCC), (2) GDC TCGA Kidney Clear Cell Carcinoma/KIRC (ccRCC) and (3) GDC TCGA Kidney Papillary Cell Carcinoma/KIRP (pRCC) (Supplemental Table S1). We then combined the clinical information, survival status and RNA-seq data for each type of RCC. Normalized RNA-seq data were converted into transcripts per million (TPM) and then transformed to log2 (TPM + 1) for chart plotting by using R language (R version 4.0.3: www.r-project.org). Our studies indicated that survivin mRNA is significantly enhanced in all three subtypes of RCC tumors in comparison with their normal counterparts (Fig. 1A). However, the dynamic profile of survivin mRNA upregulation in each RCC subtype becomes distinct when we sorted each of the patient cases into Stage 1 to Stage 4 in the 3 subtypes of RCC (Supplemental Table S1), respectively (Fig. 1BCD). Specifically, for chRCC, survivin mRNA is strikingly enhanced only in the later Stage 4 (Fig. 1B), while ccRCC and pRCC showed significant increase of survivin mRNA in early Stage 1 with further increase in late Stages 3 and 4 (Fig. 1CD). This implies that while enhanced survivin plays a role in the advanced stage of chRCC development, survivin may play a role in ccRCC and pRCC early initiation and development. Importantly, high expression of survivin in ccRCC and pRCC is significantly associated with worse patient survival (Fig. 2AC). Here, we need to discuss the data shown in Fig. 2B. Due to a small cohort of available chRCC patients, the *p*-value only approaches significance. However, after we doubled the cohort size from 32/group (cohort sizes) to 64, we got the p-value from *p* = 0.086 (*n* = 32) to *p* = 0.015 (*n* = 64). Furthermore, if we tripled the cohort size from 32 (cohort sizes) to 96, we got the p-value from *p* = 0.086 (*n* = 32) to *p* = 0.0028 (*n* = 96). Therefore, it is highly likely that high survivin expression is also significantly associated with worse chRCC patients’ survival.

### Survivin as a target for RCC therapeutics

Based on the literature, we will briefly summarize the treatment of RCC relevant to survivin as a target. Silibinin, a chemotherapeutic/chemopreventive compound isolated from the plant *Silybum marianum* (milk thistle), was shown to inhibit cell growth and induces apoptosis by downregulating survivin together with caspase activation and EGFR-ERK pathway inhibition in RCC [30]. It was also reported that vorinostat (an HDAC inhibitor) enhances the activity of temsirolimus (an mTOR inhibitor) in a panel of RCC cell lines as well as RCC xenografts through suppression of survivin levels [31]. These authors demonstrated that the synergistic effect of temsirolimus with vorinostat on cell viability, colonogenic survival inhibition and apoptosis induction was due to the synergistic inhibition of survivin expression [31]. Similar results and observations were derived by using the survivin inhibitor YM155 (instead of vorinostat) with termsirolimus [32]. These authors demonstrated that the effectiveness of the dual survivin/mTOR inhibition strategy was mediated by decreasing survivin levels with the corresponding induction of apoptosis [32]. The authors proposed that survivin inhibition as a novel approach to improve RCC therapy warrants further investigation [32]. However, use of YM155 to address survivin’s role in RCC treatment resistance obtained inconsistent observations [33, 34]. Whether such inconsistency is in part due to YM155 not being a survivin-specific inhibitor or due to the study methods used would need further investigation. Nevertheless, comprehensive studies of the potential use of survivin as a target for cancer therapeutics were recently reviewed elsewhere [35].

## XIAP

There are many studies on XIAP (X-linked Inhibitor of apoptosis) as a target and prognostic biomarker in other cancer types. For example, use of XIAP BIR domain as a target for discovering antagonists [36, 37]; the role of XIAP in mitochondrial membrane permeability as a target for cancer therapy [38]; and the prognostic value of XIAP in various cancer [39, 40]. In contrast, the studies of XIAP in RCC are limited. Therefore, there may be a big room for further study of XIAP in the RCC research field in the coming years. We summarize the available studies relevant to XIAP as a target and/or biomarker in RCC below.

IHC analysis of XIAP expression in 145 ccRCC indicated that XIAP protein expression was found in 95% of ccRCCs [41]. Specifically, a significant increase of XIAP expression was observed from well (G1) to poorly (G3) differentiated ccRCCs (*P* < 0.0001) and from low (pT1) to advanced (pT3) tumor stages (*P* = 0.0016). The log-rank test showed a significant inverse correlation (*p* = 0.0174) between XIAP expression and tumor aggressiveness in terms of patient survival. The multivariate Cox regression analysis revealed that XIAP expression is an independent prognostic parameter (*p* = 0.018) in ccRCC [41]. Comparable results were also obtained in other similar studies [42]. Similarly, analysis of XIAP and Smac in 66 RCC indicated a tumor stage-dependent increase of XIAP expression with a disturbed ratio of XIAP versus Smac [43]. Consistently, the use of Smac mimic peptide together with siRNA-silencing of XIAP resulted in better sensitivity of RCC cells to treatment-induced apoptosis [44]. Recently, several studies of XIAP on treatment resistance in RCC from a research group were published, and one of these studies revealed an association of XIAP with Bcl-2 family proteins Bcl-2 and Bcl-XL in RCC cells [45]. Specifically, the study indicated that siRNA-mediated XIAP silencing increased apoptosis and cytochrome C release with a rapid decrease (3 h) of Bcl-2 and Bcl-xl levels, resulting in the changes of Bcl-2/Bax and Bcl-xl/Bax ratios [45].

**Fig. 3:**
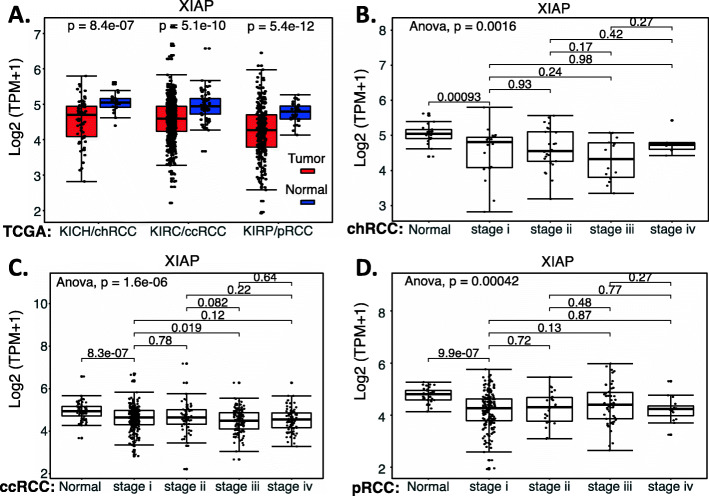
XIAP expression in RCC tumor tissues versus in the associated normal tissues: Boxplots of the XIAP expression level across TCGA-RCC subtypes in renal tumor (red) versus the associated normal renal tissues (blue) were presented (**A**). BIRC5/survivin expression among different stages of chRCC (**B**), ccRCC (**C**) and pRCC (**D**) versus the associated normal tissues was box-plotted. XIAP expression was presented in the log2 (TPM + 1) scale format. Data were presented as the mean ± SD. A t-test was used to evaluate the statistical significance of the XIAP mRNA expression level in normal renal tissues versus tumor tissues. One-way ANOVA was used to compare XIAP expression among normal renal tissues versus different stages of RCC tumor tissues. The figure was performed using R version 4.0.3

**Fig. 4:**
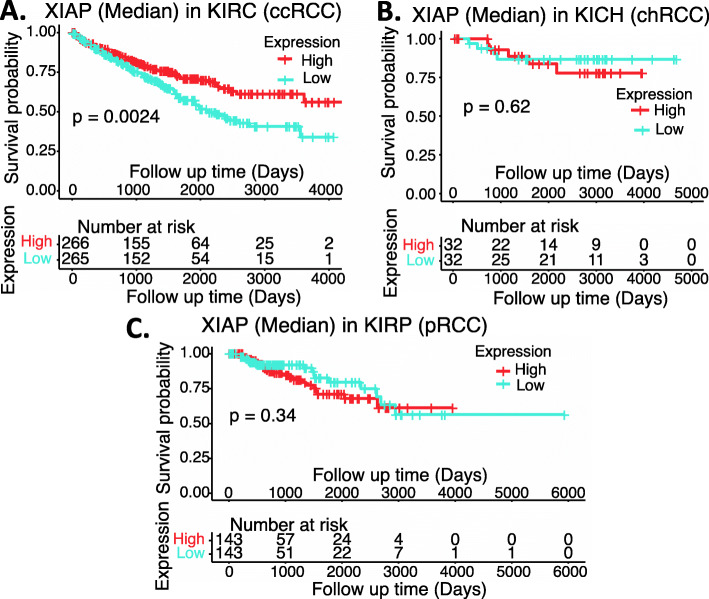
Effects of XIAP expression on RCC patient survival probability: Kaplan-Meier survival analyses of OS from TCGA-RCC cohorts of ccRCC (**A**), chRCC (**B**) and pRCC (**C**) were presented. Patients were grouped into the high XIAP expression group versus the low XIAP expression group based on the median XIAP mRNA expression. Each *p*-value for the significance from high versus low XIAP expression was calculated using the log-rank test. The figures were performed using R version 4.0.3

In order to independently evaluate the role of XIAP, we used the publicly available RCC genetic databases and performed an analysis of XIAP expression in RCC versus in normal tissues overall, as well as the matched RCC subtypes (Supplemental Table S1). We also analyzed the effect of XIAP expression on the patient survival in individual subtypes of RCC. Interestingly, XIAP was significantly decreased in RCC (Fig. 3); increased XIAP expression in ccRCC tumors is significantly associated with better ccRCC patient survival (Fig. 4A), while there is no significant difference for chRCC and pRCC patients’ survival with XIAP expression levels (Fig. 4BC). Two points may be discussed here. First, TCGA data is mRNA data-based and may not always reflect their protein expression. Second, it is also possible that while high expression of XIAP is associated with better survival in ccRCC (Fig. 4A), XIAP can still be a target for tumor elimination. This notion has been demonstrated in the case of HIF2α (see below).

## MCL-1

Use of MCL-1 (myeloid cell leukemia-1) as a biomarker and target for cancer prognosis and treatment has more publications than the publications from the use of XIAP as a biomarker and target in the cancer field. For example, in terms of MCL-1 as a prognostic biomarker, analysis of Bcl-2, Bax, and MCL-1 expression in 185 chronic lymphocytic leukemia (CLL) patients, authors found that MCL-1 expression was significantly correlated with stage of disease, lymphocyte doubling time, Ig V_H_ gene mutation status, CD38 expression, and ZAP-70 expression [46]. These authors concluded that MCL-1 is a key controller of in vitro drug resistance and is an important regulator of disease progression and outcome in CLL [46]. Recent studies also demonstrated the same concept in other cancer types [47, 48]. In terms of MCL-1 acting as a rational drug treatment therapeutic target, a genome-wide RNA interference screen identified MCL-1 as a key downstream survival factor in malignant glioma for therapeutic implications [49]. Targeting MCL-1 enhances DNA replication stress sensitivity to cancer therapy [50]. Studies on malignant plasma cells from 60 patients indicated that MCL-1 dependence significantly increased from 33% at diagnosis to 69% at relapse, suggesting a plasticity of the cellular dependency favoring MCL-1 dependencies at relapse [51]. Mechanistically, Bak is a crucial mediation of cell death induced by MCL-1 mimetic A1210477 [51]. We provide several review articles focusing on MCL-1 as a target for cancer therapeutics for further reading [52–55].

In contrast to the active MCL-1 studies in the cancer field, limited studies on MCL-1 are available in renal/kidney cancer. This may provide a good opportunity for further exploration of MCL-1 as a biomarker and target in kidney/renal cancer. We now summarize the information related to MCL-1 as a target and prognostic biomarker in RCC. It was reported that (i) RNAi-mediated silencing of MCL-1 sensitized the Bcl-2 inhibitor ABT-737 to induce apoptosis in RCC cell lines [56]; (ii) a cell-permeable pyrrazolopyrimidine derivative (compound C) enhances TRAIL-induced apoptosis in human Caki renal cancer cells by reactive oxygen species (ROS)-mediated c-FLIP(L) and MCL-1 downregulation [57], and (iii) similar results were also found with anisomycin [58], dicoumarol [59], curcumin [60], YM155 [61] and the histone lysine-specific demethylase 1 inhibitor, SP2509 [62] in RCC cells. It was also reported that Ras association domain family member 6 (RASSF6) as a tumor suppressor inhibits sorafenib resistance by repressing MCL-1 through the JNK-dependent pathway in RCC cells [63]. However, we found that one publication is inconsistent with these findings summarized above and stated that Long noncoding RNA, linc-ITGB1 promotes migration and invasion of ccRCC by downregulating MCL-1 [64]. Based on Fig. 4 (relevant to MCL-1) data shown in [64], lentivirus-mediated overexpression of linc-ITGB1 indeed decreased the endogenous MCL-1 expression. However, while the MCL-1 expression data derived from ccRCC tissues showed a striking increase when compared with adjacent tissue (their Fig. 4C), these authors still stated that “MCL-1 was significantly downregulated in ccRCC tissues compared with adjacent tissues.” [64]. Furthermore, if the statement is a typo error, then based on the presented data (their Figs. 1C, 4C), both linc-ITGB1 and MCL-1 were highly expressed in the clinical ccRCC tissue cohort used, instead of an opposing relationship of linc-ITGB1 expression versus MCL-1 expression as these authors claimed [64]. It is our assessment that the role of linc-ITGB1 overexpression-associated downregulation of MCL-1 in ccRCC cells in their studies require further investigation.

**Fig. 5:**
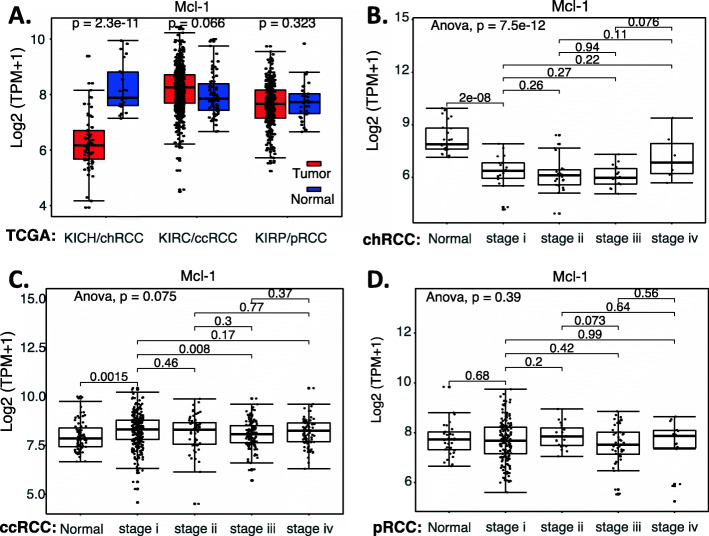
MCL-1 expression in RCC tumor tissues versus in the associate normal tissues: Boxplots of the MCL-1 expression level across TCGA-RCC subtypes in renal tumor (red) versus the associated normal renal tissues (blue) were presented (**A**). MCL-1 expression among different stages of chRCC (**B**), ccRCC (**C**) and pRCC (**D**) versus the associated normal tissues was box-plotted. MCL-1 expression was presented in the log2 (TPM + 1) scale format. Data were presented as the mean ± SD. A t-test was used to evaluate the statistical significance of the mRNA expression level in normal renal tissues versus tumor tissues. One-way ANOVA was used to compare MCL-1 expression among normal renal tissues versus different stages of RCC tumor tissues. The figure was performed using R version 4.0.3

**Fig. 6:**
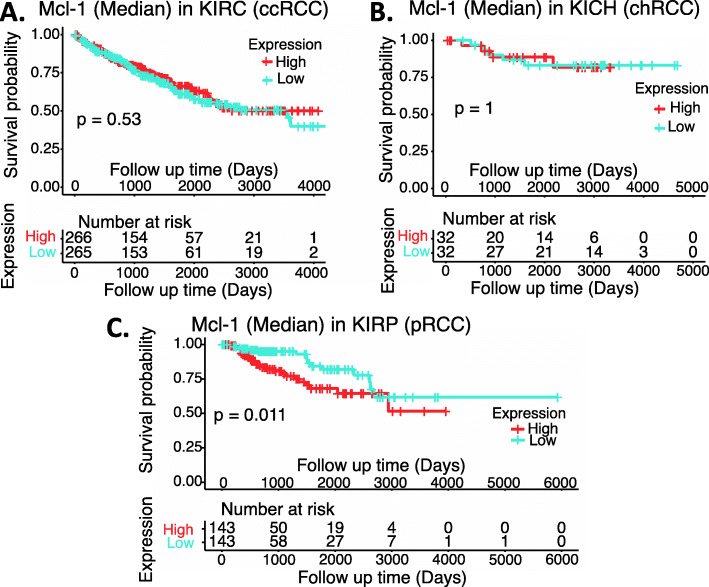
Effects of MCL-1 expression on RCC patient survival probability: Kaplan-Meier survival analyses of OS from TCGA-RCC cohorts of ccRCC (**A**), chRCC (**B**) and pRCC (**C**) were presented. Patients were grouped into the high MCL-1 expression group versus the low MCL-1 expression group based on the median MCL-1 mRNA expression. Each *p*-value for its significance from high versus low MCL-1 expression was calculated using the log-rank test. The figures were performed using R version 4.0.3

Based on our review of the publications related to RCC, a strong role of MCL-1 as a target and/or prognostic biomarker in RCC has not been established. Therefore, we retrieved the publicly available RCC versus normal tissue datasets (Supplemental Table S1) and performed an analysis of MCL-1 expression overall in RCC versus in normal tissues as well as in the major subtypes of RCC in different stages versus in normal tissues. The data showed that MCL-1 expression in chRCC is significantly decreased (Fig. 5AB), while there is no significant difference for MCL-1 expression in ccRCC and pRCC (Fig. 5ACD). We then analyzed the survival association of RCC patients with MCL-1 expression in RCC patients’ tumors versus normal tissues. We found that while the expression level of MCL-1 in ccRCC and chRCC tumors does not affect patients’ survival (Fig. 6AB), high expression of MCL-1 in pRCC tumors appears to be associated with worse patient survival (Fig. 6C).

## HIF1α and HIF2α

In normal renal cells, pVHL (von Hippel-Lindau, a tumor suppressor protein, acting as an E3 ubiquitin ligase) binds to and degrades HIF (hypoxia inducible factor/hypoxia induced factor). However, at least in most ccRCC tumor cases, VHL/pVHL is mutated and loses its function and thus, ccRCC cells become pVHL-deficient. As a result, HIF proteins are overexpressed in ccRCC. There are a lot of high impact publications around the VHL-HIF pathway [65–68]. However, a detailed review to update the VHL-HIF-relevant pathway is not the focus of this review. Instead, we will focus on HIF1α and HIF2α as biomarkers and/or targets for RCC tumor potential therapeutics, which is a popular area that has been growing in the past decade or so.

### HIF1α and/or HIF2α as biomarker(s) and target(s) in RCC

Literature review generally indicated contradictive roles and properties for HIF1α versus HIF2α in RCC. For example, [1] the expression of HIF1α and HIF2α are mutually suppressed, and while HIF1α retards RCC tumor growth, HIF2α enhances RCC tumor growth [69]. However, it was also reported that ccRCC patients with high HIF1α expression (> 35%) had significantly worse survival than patients with low HIF1α expression (≤35%) (median survival, 13.5 versus 24.4 months, respectively) [70]. pVHL-deficient ccRCC tumors strongly expresses both HIF1α and HIF2α, chRCC tumors predominantly express HIF2α with weaker HIF1α expression, and half of pRCC tumors express HIF2α without expressing HIF1α [71]. Interestingly, using an autochthonous ccRCC mouse model, a recent study demonstrated that HIF1α is essential for tumor formation whereas HIF2α deletion has only minor effects on tumor initiation and growth in mouse model [72]. The authors conclude that both HIF1α and HIF2α are required for the clear cell phenotype [72]. Their transcriptomic and proteomic analyses reveal that HIF1α regulates glycolysis, while HIF2α regulates genes associated with lipoprotein metabolism, ribosome biogenesis, E2F and Myc transcriptional activities [72]. HIF2α-deficient tumors are characterized by increased antigen presentation, interferon signaling and CD8(+) T cell infiltration and activation, while single copy loss of HIF1α or high levels of HIF2α mRNA expression correlate with altered immune microenvironments in human ccRCC [72]. However, researchers may want to keep in mind that mouse ccRCC tumor models may not always fully reflect human ccRCC (see additional studies in the next paragraph).

In terms of biomarkers, one study used 94 ccRCC tumor tissues to immunochemically determine the expression of nuclear HIF1α versus cytoplasmic HIF1α. The study revealed that overexpression of cytoplasmic HIF1α is associated with a higher nuclear grade, larger tumor size, higher stage and shorter survival, while nuclear HIF1α overexpression is associated with better diagnostic parameters (i.e., lower nuclear grade, smaller tumor size and longer survival) [73]. However, a recent comprehensive study revealed that macrophage HIF1α appears to be an independent prognostic indicator for ccRCC [74]. These authors used a large cohort of ccRCC tumor tissues (380 on a tissue microarray, an additional 57 ccRCC from patients treated with antiangiogenic therapy for response associations analysis) to assess the expression of HAF (hypoxia-associated factor, which regulates HIFs), HIF1α and HIF2α in ccRCC. The study revealed that HIF1α was primarily expressed in tumor-associated macrophages (TAM), whereas HIF2α and HAF were mainly expressed in tumor cells [74]. TAM-associated HIF1α was significantly associated with high tumor grade and increased metastasis and was independently associated with decreased overall survival [74]. Furthermore, elevated TAM HIF1α was significantly associated with resistance to antiangiogenic therapy. In contrast, high HAF or HIF2α were associated with low grade, decreased metastasis, and increased overall survival [74]. These authors concluded that their findings highlight a potential role of TAM HIF1α in ccRCC progression and support the reevaluation of HIF1α as a therapeutic target and marker of disease progression [74]. However, it was also reported that high nuclear HIF2α expression is associated with smaller tumor sizes and lower Fuhrman grades, whereas ccRCC tumors with high cytoplasmic HIF2α more often had positive lymph nodes, distant metastases and higher Fuhrman grades [75]. The data indicated that cytoplasmic variables remain significant predictors of cancer specific survival, while neither nuclear HIF2α variables are retained [75]. Similar results were obtained in a recent study that revealed that ccRCC with high (cytoplasmic) HIF2α expression is associated with unfavorable disease [76].

**Fig. 7:**
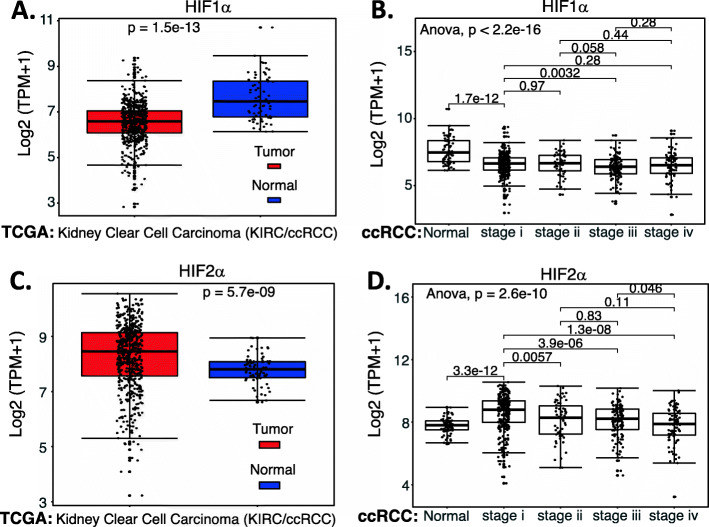
Expression of HIF1α and HIF2α in ccRCC tumor tissues versus in the associated normal tissues: The expression level of HIF1α (**A**) or HIF2α (**C**) across TCGA-RCC subtypes in renal tumor (red) versus the associated normal renal tissues (blue) were box-plotted. The expression of HIF1α (**B**) or HIF2α (**D**) among different stages of ccRCC tumor tissues versus the associated normal tissues was box-plotted. The expression of HIF1α or HIF2α was presented in the log2 (TPM + 1) scale format. Data were presented as the mean ± SD. A t-test was used to evaluate the statistical significance of the mRNA expression level in normal renal tissues versus tumor tissues. One-way ANOVA was used to compare HIF1α expression or HIF2α expression among normal renal tissues versus different stages of ccRCC tumor tissues. The figure was performed using R version 4.0.3

**Fig. 8:**
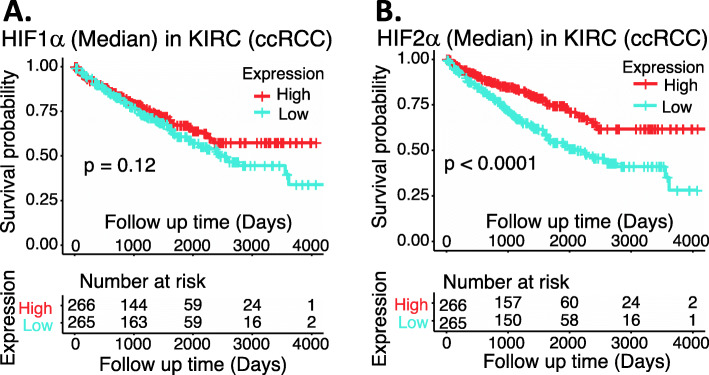
Effects of HIF1α and HIF2α expression on ccRCC patient survival probability: Kaplan-Meier survival analyses of patient OS from the TCGA-ccRCC cohorts of ccRCC for HIF1α (**A**) or for HIF2α (**B**) were presented. **A** ccRCC patients were grouped into the high HIF1α expression group versus the low HIF1α expression group based on the median HIF1α mRNA expression. **B** ccRCC patients were grouped into the high HIF2α expression group versus the low HIF2α expression group based on the median HIF2α mRNA expression. Each *p*-value for its significance from the high expression versus low expression of HIF1α (**A**) or HIF2α (**B**) was calculated using the log-rank test. The figures were performed using R version 4.0.3

Based on the Supplemental Table S1 datasets, we performed an analysis of HIF1α and HIF2α expression in ccRCC (data not available for chRCC and pRCC) versus normal tissues as well as in the different stages versus in normal tissues. The data indicated that HIF1α expression is decreased (Fig. 7A) and HIF2α is increased (Fig. 7C), and both happened in the Stage 1 of ccRCC/KIRC (Fig. 7BD). Interestingly, while HIF1α and HIF2α expression in ccRCC exhibited an opposed behavior (Fig. 7), the increased expression of either HIF1α or HIF2α in ccRCC appears to be associated with a favorable patient survival rate (Fig. 8), although in the case of HIF1α, it is not significant (Fig. 8A). However, we need to keep in mind that TCGA data is mRNA-based data and may not always reflect their protein expression. Importantly, while high expression of HIF2α is associated with better survival in ccRCC (Fig. 8B), studies demonstrated that HIF2α is a good target for elimination of the ccRCC tumor (see the section below in detail).

Together, there is some inconsistency in the literature in terms of HIF1α and HIF2α acting as RCC favorable or unfavorable biomarker(s) and target(s). Generally, majority of the available studies have identified that HIF1α plays an inhibitory role and HIF2α plays an oncogenic role in promoting RCC/ccRCC malignancy. It appears that HIF2α would be a good target at least for ccRCC tumor therapeutics. This view is consistent with the conclusion from a recent review article [77], although some inconsistent with the data shown in Fig. 8.

### Target HIF2α for ccRCC tumor therapeutics

**Fig. 9:**
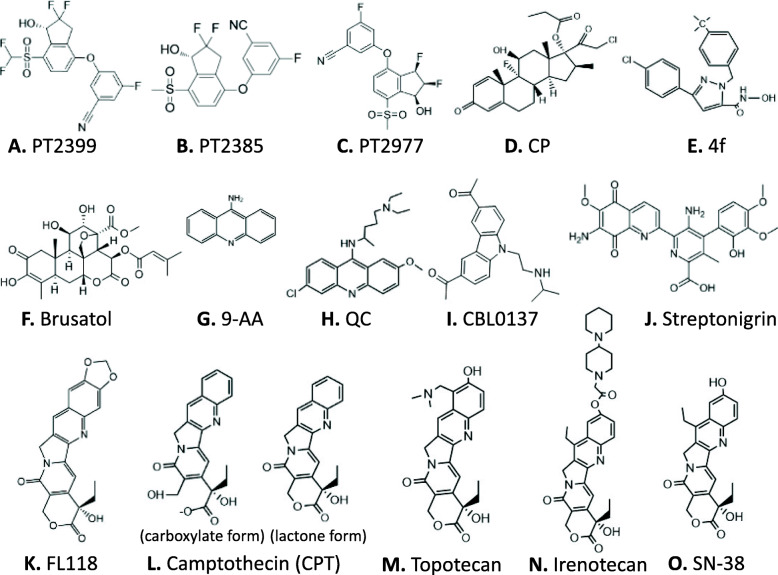
Chemical structures of the compounds that have been discussed in this article: Each compound structure was either generated using the ChemDraw Prime 16 software (Perkin Elmer) or downloaded from appropriate online sources

There are some inconsistent observations in terms of HIF2α as a human ccRCC therapeutic target (reviewed above). However, the cornerstone is that use of HIF2α as a drug target resulted in promising progression. Cho et al. showed that a small molecule named PT2399 (Fig. 9A) directly binds and inhibits HIF2α, which causes tumor regression in an on-target fashion in preclinical mouse models established from human primary and metastatic pVHL-defective ccRCC [78]. Another study demonstrated that PT2399 dissociated HIF2 (an obligatory heterodimer of HIF2α-HIF1β) in human ccRCC cells and suppressed tumorigenesis in 56% (10 out of 18) of such lines [79]. However, these authors found that some VHL-mutant ccRCCs were resistant to PT2399, despite HIF2 dissociation in tumors and that HIF2 inhibition in mice with prolonged PT2399 treatment led to resistance [79]. Together, these two studies validated HIF2α [78] and HIF2 [79] as PT2399’s target, respectively, in human ccRCC even though some ccRCCs are HIF2 independent [79].

A third study from the Peloton Therapeutics group described an orally active small-molecule inhibitor named PT2385, which is structurally similar to PT2399 (Fig. 9B), as a specific antagonist of HIF2α, which can allosterically block PT2385 dimerization with the HIF1α/2α transcriptional partners, aryl hydrocarbon receptor nuclear translocator (ARNT)/HIF1β [80]. PT2385 inhibited the expression of HIF2α-dependent genes, including VEGF-A, PAI-1, and cyclin D1 in ccRCC cell lines and tumor xenografts [80]; and treatment of tumor-bearing mice with PT2385 caused tumor regressions, suggesting HIF2α is a pivotal oncogenic driver of human ccRCC [80]. The synthetic design and identification of PT2385 can be found in a more recent publication [81]. Subsequently, a phase 1 dose-escalation trial of PT2385 in patients with previously treated advanced ccRCC was published [82]. PT2385 was administered orally at twice-per-day with doses of 100 to 1800 mg, according to a 3 + 3 dose-escalation design in 26 patients, followed by an expansion phase with 25 patients at the recommended phase 2 dose (RP2D). No dose-limiting toxicity was observed at any doses. Based on safety, pharmacokinetics, and pharmacodynamics, the RP2D was defined as 800 mg twice per day. PT2385 was well tolerated, with anemia (grade 1 to 2, 35%; grade 3, 10%), peripheral edema (grade 1 to 2, 37%; grade 3, 2%), and fatigue (grade 1 to 2, 37%; no grade 3 or 4) being the most common treatment-emergent adverse events [82]. Complete response, partial response, and stable disease as the best response were achieved by 2, 12, and 52% of patients, respectively [82]. Furthermore, a follow-up study showed that PT2385 inhibited HIF2 in nontumor tissues in all but one patient, who had the lowest drug concentrations; PT2385 dissociated HIF2 complexes even in ccRCC metastases, and inhibited HIF2 target gene expression; in contrast, HIF1 complexes were unaffected [83]. However, prolonged PT2385 treatment resulted in the acquisition of resistance; and these authors identified a gatekeeper mutation (G323E) in HIF2α, which interferes with drug binding and precluded HIF2 complex dissociation [83]. In addition, they also identified an acquired TP53/p53 mutation elsewhere, suggesting a possible alternative mechanism of resistance [83].

Recently, another PT2385 analog named PT2977 (Fig. 9C) was reported from the Peloton Therapeutics group [84]. This is because PT2385 was restricted by variable and dose-limited pharmacokinetics resulting from extensive in vivo metabolism of PT2385 into the glucuronide metabolite. Therefore, these researchers developed the second generation of the HIF2α inhibitor PT2977 with decreased lipophilicity [84]. PT2977 increased potency and improved pharmacokinetic profile via reducing in vivo metabolism of PT2977 to glucuronide metabolite. In a phase 1 dose-escalation study, the clinical pharmacokinetics for PT2977 support the hypothesis that attenuating the glucuronidation rate would improve exposure and reduce variability in patients. Evidence from PT2977 clinical activity supported further studies in the treatment of ccRCC [84].

Finally, for those researchers who would like to have additional reading on the regulation and potential therapeutics for ccRCC tumors, a recent review article would be a good starting point [85].

## NRF2

### Role of NRF2 as a biomarker and a target in cancer resistance and treatment

NRF2 (Nuclear factor erythroid 2-related factor 2) is a transcription factor and can increase the production of several antioxidant enzymes that can eliminate reactive oxygen species (ROS, refer to the Supplemental Material 1 for more information). Cancer cells use multiple signaling pathways to constitutively maintain a tolerable level of ROS for their malignancy. Many anticancer drugs can rapidly induce ROS overproduction in cancer cells to kill the cells. Therefore, in terms of cancer treatment, activation of NRF2 is a treatment resistance mechanism [86]. Additionally, NRF2 activates oncogenes that are unrelated to ROS elimination [87]. Therefore, NRF2 activation in cancer cells can not only neutralize ROS production but also activate other oncogenic and treatment-resistant proteins to increase cancer cell survival and tumor resistance to treatment. NRF2 activation is a hallmark of cancer [88] and a potential cancer therapeutic target [89–91] as well as a biomarker of cancer malignancy for prognosis and treatment. For example, NRF2 activation promotes lung cancer metastasis [92] and associates with poor clinical outcomes [93]; enhanced NRF2 expression increases risk of high tumor mutation burden in the genome overall in acute myeloid leukemia [94] and correlates with poor prognosis in colorectal cancer patients [95]; upregulation of NRF2 expression induced by Keap1 downregulation contributes to poor prognosis and Axitinib resistance in RCC [96]; and the NRF2/HO-1 axis can be a prognostic factor in ccRCC [97]. It is important that at least a part of these NRF2 functions may be involved in its role in maintaining cancer stem cell survival [98].

**Fig. 10:**
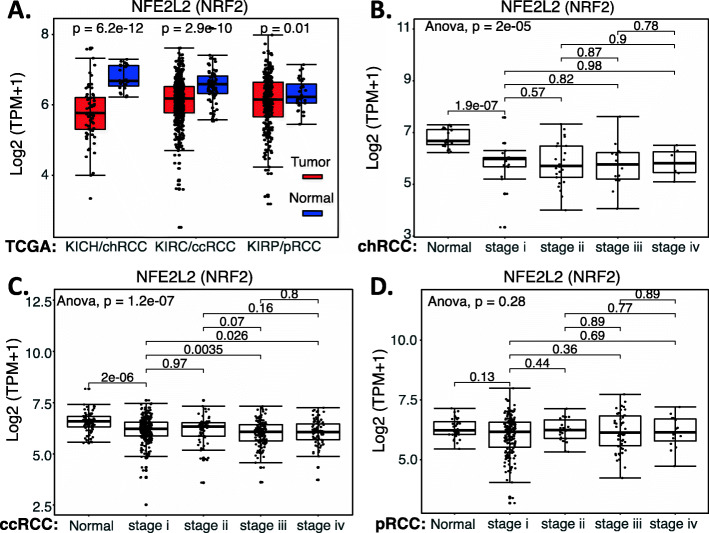
NRF2 expression in RCC tumor tissues versus in the associated normal tissues: Boxplots of the NRF2 expression level across TCGA-RCC subtypes in renal tumor (red) versus the associated normal renal tissues (blue) were presented (**A**). NRF2 expression among different stages of chRCC (**B**), ccRCC (**C**) and pRCC (**D**) versus the associated normal renal tissues was box-plotted. NRF2 expression was presented in the log2 (TPM + 1) scale format. Data were presented as the mean ± SD. A t-test was used to evaluate the statistical significance of the NRF2 mRNA expression level in normal renal tissues versus tumor tissues. One-way ANOVA was used to compare NRF2 expression among normal renal tissues versus different stages of RCC tumor tissues. The figure was performed using R version 4.0.3

**Fig. 11:**
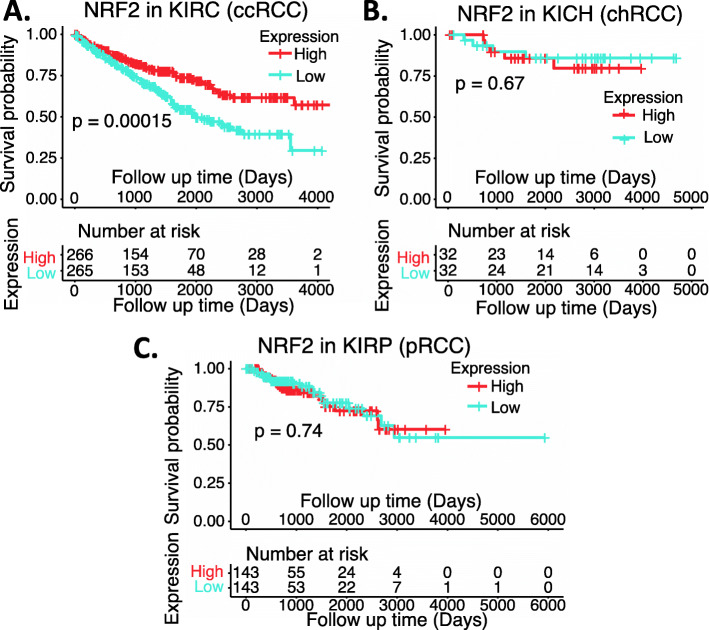
Effects of NRF2 expression on ccRCC patient survival probability: Kaplan-Meier survival analyses of OS from TCGA-RCC cohorts of ccRCC (**A**), chRCC (**B**) and pRCC (**C**) were presented. Patients were grouped into the high NRF2 expression group versus the low NRF2 expression group based on the median NRF2 mRNA expression level. Each *p*-value for the significance from high versus low NRF2 expression was calculated using the log-rank test. The figures were performed using R version 4.0.3

We collected the NRF2 expression data available from public domains and compared the expression of NRF2 in RCC versus normal tissues as well as the survival association of RCC patients with high NRF2 expression versus with low NRF2 expression. Our analyzed data indicated that NRF2 expression is significantly decreased in chRCC and ccRCC overall (Fig. 10A) or in matched disease stage 1 to 4 (Fig. 10BC) in comparison with normal tissue (Fig. 10ABC). However, although NRF2 expression showed a significant decrease in pRCC overall (Fig. 10A), there is no significant NRF2 expression in the disease-matched stage 1 to 4 in comparison with normal tissue (Fig. 10D). Intriguingly, only in ccRCC, high NRF2 expression is associated with better patient survival (Fig. 11A), while the expression level of NRF2 in chRCC and pRCC exhibit no differential association with patient survival (Fig. 11BC). Again, TCGA data is mRNA data-based, which may not always reflect the protein expression level. Additionally, as demonstrated in the case of HIF2α, it is also possible that while the high expression of NRF2 is associated with better survival in ccRCC, NRF2 can still be a target for tumor elimination.

### Role of NRF2 in hereditary leiomyomatosis RCC

As described earlier, RCC can be classified into ccRCC (70–80%), pRCC (15–20%), chRCC (5–10%) and cdRCC (< 1%) [1, 2]. While patients with ccRCC cancer have many options for treatment, either using chemotherapies [99] and/or targeted therapies [100, 101]; drugs that are specifically aimed at treatment of pRCC cancer remain to be developed. The pRCC can be classified into Type 1 and Type 2 [1]. Type-2 pRCC is highly aggressive, is treatment resistant and can be sub-classified into four subtypes: (1) CDKN2A silencing, (2) chromatin modifier SETD2 mutations, (3) TFE3 fusions, and (4) activation of NRF2-antioxidant response element (ARE) pathway [1]. The hereditary leiomyomatosis RCC (HLRCC) belongs to the last subtype. The affected individuals are at risk of developing aggressive pRCC due to the mutation of the fumarate hydratase (FH) gene [102, 103]. The mutated FH results in tricarboxylic acid (TCA, Krebs) cycle deficiency, leading to both overproduction of ROS and high expression of NRF2 for countering ROS overproduction-resulted cancer cell killing. NRF2 activation plays a critical role in the treatment resistance of FH mutation-induced type-2 pRCC (FHpRCC) tumor cells in addition to the high expression of XIAP, MDM2, p-Akt, etc. Metastatic pRCC continues to have limited treatment options [104]. While certain promising results exist with some treatments [105], better options are urgently needed for targeting this particular subset of the patient population. This is challenging, but it is also a great opportunity for the research community in the coming years. Nevertheless, we have sorted pRCC tissue samples and normal tissues into Type 1 and Type 2 pRCC (Supplemental Table S3), the expression of NRF2 in Type 1 pRCC has no significant difference, and in Type 2 pRCC has a marginal significance of NRF2 expression decrease in tumor tissues versus normal tissues (Supplemental Figure S1A). However, after matching to disease stages, no significant differences were found in either Type 1 pRCC or Type 2 pRCC in tumor tissues versus in normal tissues (Supplemental Figure S1BCD). Interestingly, after sorting NRF2 expression into high and low categories among Type 1 pRCC and Type 2 pRCC for patient survival analysis, we found that while there is no significant difference for patients with Type 1 pRCC, there is a potentially significant difference for patients with Type 2 pRCC (Supplemental Figure S2). When we doubled and tripled the Type 2 pRCC cohort sizes, the *p*-value was changed from 0.14 to 0.037 and to 0.01 (Supplemental Figure S2B). This result is consistent with the defined role of NRF2 in Type 2 pRCC sub type (FHpRCC).

### NRF2 antagonists/inhibitors

In comparison with the research focusing on the Keap1 inhibitors (i.e., Keap1-NRF2 disruptors/NRF2activators) and NRF2 agonists/activators, the research area of NRF2 antagonists/inhibitors for cancer treatment lags behind with very limited publications, especially concerning RCC. Of course, this also opens a great opportunity in the coming years for researchers. As of 2017, there are no NRF2 inhibitors that are clinically available. Choi, et al. screened ~ 4000 clinical compounds and found that clobetasol propionate (CP, Fig. 9D) is the most potent NRF2 inhibitor [106]. Mechanistically, CP prevented NRF2 nuclear accumulation and promoted β-TrCP-dependent NRF2 degradation in a glucocorticoid receptor- and a glycogen synthase kinase 3 (GSK3)-dependent manner [106]. As a result, CP induced oxidative stress and strongly suppressed the anchorage-independent growth in Keap1-mutated tumors, but not in wild-type KEAP1 tumors [106]. Furthermore, CP alone or in combination with rapamycin strongly inhibited the in vitro and in vivo growth of tumors harboring mutations in Keap1 or both Keap1 and Lkb1 frequently observed in lung cancer [106]. These authors proposed that the use of CP alone or in combination with rapamycin could be a potential therapeutic strategy for tumors harboring both KEAP1 and LKB1 mutations [106]. Another example is the discovery of a novel pyrazolyl hydroxamic acid derivative, 4f (Fig. 9E) that inhibits NRF2 activity [107]. 4f downregulated NRF2 protein, had a profound growth-inhibitory effect on all three acute myeloid leukemia (AML) cell lines tested (THP-1, HL-60 and U937), and induced apoptosis, which was evidenced by flow cytometry, caspase-2 cleavage and PARP cleavage [107]. Furthermore, upregulation of NRF2 by tert-butylhydroquinone (tBHQ) or overexpression of NRF2 could ameliorate 4f-induced growth inhibition and apoptosis [107]. Interestingly, 4f could reduce Bcl-2 expression, change Bcl-2/Bax ratio, and induce apoptosis, at least in part, via mitochondrial-dependent signaling [107].

To date, the most studied NRF2 inhibitor is brusatol (Fig. 9F). For example, Xiang et al. reported that brusatol abrogate gemcitabine-induced NRF2 activation in pancreatic cancer cells and potentiates gemcitabine-induced cell growth inhibition in vitro and xenograft tumor growth inhibition in vivo with reduced NRF2 expression in brusatol-treated xenograft tumors [108]; Yang, et al. reported that brusatol synergistically enhanced the antitumor activity of trastuzumab against HER2-positive cancer cells [109]; trastuzumab markedly enhanced brusatol-induced ROS accumulation and apoptosis level [109]. The authors stated that the study is a new insight on exploring NRF2 inhibition in combination with HER2-targeted trastuzumab as a potential clinical treatment regimen for treating HER2-positive cancers [109]; Very recently, Xie, et al. reported that in lung cancer cells, brusatol significantly suppressed the expression of NRF2 and HO-1 (a NRF2 downstream target), and abrogated tBHQ-induced NRF2 activation [110]; brusatol suppressed the expression level of Bcl-2 and Bcl-xl, accentuated Bax and Bak, increased cleaved caspases-3/8, and cleaved PARP but upregulated XIAP [110]. These authors proposed that brusatol action may involve the modulation of ROS-mediated mitochondrial-dependent pathway and inhibition of NRF2-mediated antioxidant response [110]. For additional past studies, the reader may refer to the recent summary paper [111].

### Significance of NRF2 mutation

A recent publication documented the somatic NRF2 gain-of-function mutations in cancer [112]. However, mutation of either Keap1 and NRF2 in RCC is a very rare event (Supplemental Table S2) and the mutant cohorts were too small to form meaningful evaluation of the role of their mutations in RCC. We now provide several relevant publications that are worthy of further reading [113–115].

## MDM2 (HDM2) in RCC

A PubMed search on March 21, 2021, revealed that there are 3675 publications having MDM2 or HDM2 in the publication title. If excluding both the key word of “p53” and “TP53” in the title, there were still resulting 1650 publications. In contrast, in the renal/kidney cancer area, the former criteria only resulted in 20 publications, with only 8 publications matching the latter criteria (i.e., without p53 or TP53 in the title). This means that there is only one publication in the renal/kidney cancer area out of every 184 publications in the PubMed. In this section, we focus on the 8 most relevant publications in renal cancer. The remaining 12 publications focused on renal cancer will be discussed in the next p53/TP53 section if appropriate.

### MDM2/Hdm2 expression as a biomarker and other potential functions in RCC

Genotyping single nucleotide polymorphisms (SNP) of MDM2-SNP309 in 200 human RCC samples versus samples from 200 age/gender-matched healthy subjects (followed by direct DNA sequencing confirmation) indicated that a significant increase in the GG genotype of the MDM2-SNP309 was observed in RCC patients compared with healthy controls [116]. IHC studies revealed that the frequency of MDM2 expression in RCC patients with GG genotypes (5 of 10, 50%) was significantly higher than that of RCC patients with TT genotypes (2 of 15, 13%) and TG genotypes (4 of 15, 26%) [116]. In contrast, the same analyses for the polymorphisms of p53-Arg72Pro and p21-Ser31Arg did not show significant association with RCC [116]. Univariate and multivariate analysis indicated that the MDM2-SNP309 GG genotype is independently associated with poor prognosis; Kaplan-Meier curve analysis showed that survival of RCC patients with GG carriers was significantly worse than that of RCC patients with TG + TT genotypes [116]. Together, the study implies that increased MDM2 expression is associated with increased risk of developing RCC and the MDM2 polymorphism is an independent adverse prognostic factor for RCC. That is, RCC patients with the MDM2-309GG genotype may have worse prognosis and lower survival.

A similar but distinct finding was reported from the use of an unselected German cohort of 197 consecutive RCC patients [117]. Among this RCC cohort, the GG, GT and TT variants were detected in turn in 18/197 (GG, 9.1%), 116/197 (GT, 58.9%) and 63/197 (TT, 32.0%) RCC patients [117]. Interestingly, this study indicated that there is no association between age at tumor onset and MDM2-SNP309 genotypes from the analysis of the entire RCC cohort or among the male RCC patients, the female GG patients (median age 59.5 years) were diagnosed 13.5 years earlier than the TT females (median age 73 years) [117]. In order to further study the age dependency of tumor onset, a second, age-selected cohort of 205 RCC patients was investigated, and the result indicated that (1) the GG type occurs more often at lower tumor stages and tumor grades compared with higher stages; and (2) while the percentage of the GG variant was only slightly higher in the female younger age group, the percentage of the GG variant was remarkably higher in the male younger age group versus the old age group (19.4% vs 8.0%) [117]. These authors concluded that female Caucasian RCC patients with the MDM2-SNP309 GG genotype have significantly earlier tumor onset than patients with the wild-type TT genotype [117].

It is well known that MDM2 is an E3 ligase oncogenic protein and is a negative regulator of wild type p53 in cancer cells by direct ubiquitination of p53 for proteosome-mediated p53 degradation (of note, mutant p53 is out of MDM2 control and this is consistent with the fact that mutant p53 has a high expression level in cancer cells). Therefore, enhanced MDM2 expression could be one signal for cancer malignancy. Several interesting studies relevant to this topic are discussed here. IHC evaluation revealed that MDM2 protein expression increased stepwise throughout every steps of metastasis/recurrence in the two cases of Renal Epithelioid Angiomyolipoma (EAML) studied, although it was negative in primary tumors [118]. This study suggests that MDM2 could play an important role in the recurrence/metastasis of renal EAML. IHC studies in an advanced type 1 sarcomatoid pRCC showed MDM2 expression and amplification [119]. A recent report indicated that while p53 stability in RCC was inversely related to the expression level of MDM2 and Transglutaminase 2 (TGase2, a protein involved in autophagic protein degradation), inhibition of TGase2 but not MDM2 in an in vivo RCC model had efficient anticancer effects [120]. However, MDM2 acting as an oncogenic protein may play additional roles besides controlling wild type p53. It was shown that the oncogene MDM2/Hdm2 is implicated in the regulation of the transcription factor, HIF1; and the siRNA-mediated downregulation of MDM2 decreased the expression of HIF1α and HIF2α in VHL-defective RCC [121]. The same research group also found that in RCC cells, siRNA ablation of MDM2/Hdm2 leads to increasing VEGF and PAI-1 proteins but decreasing ET-1 [121]. The effect is independent of VHL and p53 but dependent on MDM2 ablation and the phosphorylation of ERK1/2 [121]. MDM2 such effects on VEGF, PAI-1 and ET-1 can be reversed by adding the MAP/ERK1/2 kinase inhibitors PD98059 and PD184352 [121]. Additional information on MDM2 will be presented in the p53/TP53 section below.

**Fig. 12:**
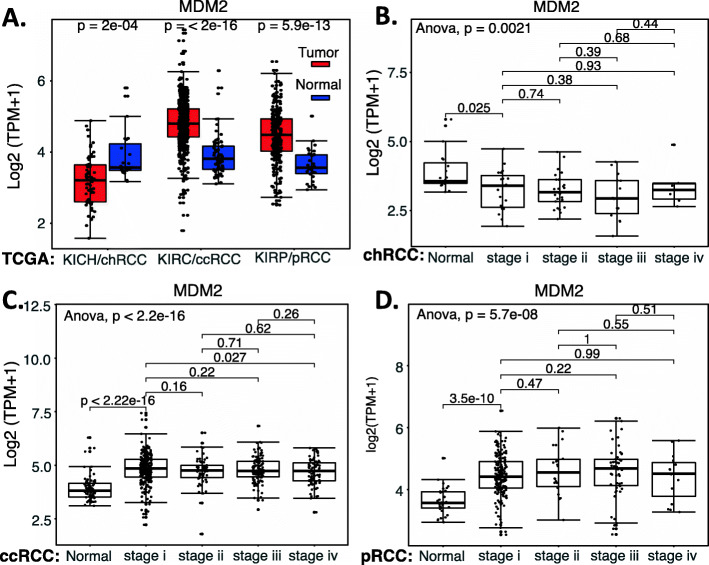
MDM2 expression in RCC tumor tissues versus in the associated normal tissue: Boxplots of the MDM2 expression level across TCGA-RCC subtypes in renal tumor (red) versus the associated normal renal tissues (blue) were presented (**A**). MDM2 expression among different stages of chRCC (**B**), ccRCC (**C**) and pRCC (**D**) versus the associated normal tissues was box-plotted. MDM2 expression was presented in the log2 (TPM + 1) scale format. Data were presented as the mean ± SD. A t-test was used to evaluate the statistical significance of the MDM2 mRNA expression level in normal renal tissues versus tumor tissues. One-way ANOVA was used to compare MDM2 expression among normal renal tissues versus different stages of RCC tumor tissues. The figure was performed using R version 4.0.3

**Fig. 13:**
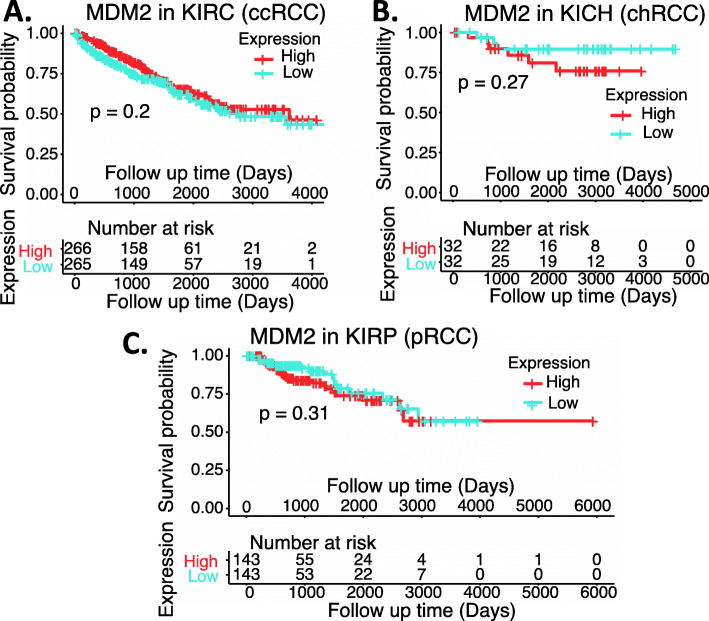
Effects of MDM2 expression on RCC patient survival probability: Kaplan-Meier survival analyses of OS from TCGA-RCC cohorts of ccRCC (**A**), chRCC (**B**) and pRCC (**C**) were presented. Patients were grouped into the high MDM2 expression group versus the low MDM2 expression group based on the median MDM2 mRNA expression. Each *p*-value for its significance from high versus low MDM2 expression was calculated using the log-rank test. The figures were performed using R version 4.0.3

We performed a comparison of MDM2 expression in RCC tumor tissues versus normal tissues as well as a survival association analysis of RCC patients with MDM2 expression (high versus low). Our analysis indicated that while MDM2 significantly decreased from early stage 1 in chRCC (Fig. 12AB) in comparison with normal tissues, MDM2 significantly increased from early stage 1 in both ccRCC and pRCC (Fig. 12ACD). However, the significance of such a unique pattern of MDM2 expression modulation in the three major RCC types is unclear, because when we sorted patients into those with high MDM2 expression versus those with low MDM2 expression, the patient survival analysis indicated that the expression level of MDM2 in RCC is not associated with patient survival (Fig. 13). However, this may not necessarily mean that MDM2 cannot be used as a target for tumor growth inhibition. As demonstrated in the case of HIF2α, while high expression HIF2α is associated with better survival in ccRCC, HIF2α was demonstrated to be a good target for ccRCC tumor elimination.

Finally, as of March 2021, only two papers describe MDM4/MDMX in RCC. One found that miR-33a inhibits cell growth in renal cancer by downregulation of MDM4 expression [122], and the other found that long non-coding RNA SNHG12 functions as a competing endogenous RNA to regulate MDM4 expression by sponging miR-129-5p in ccRCC [123]. Therefore, we performed an analysis of Mdm4 expression in RCC tumor tissues versus in normal tissues as well as a survival association analysis of the RCC patients with high Mdm4 expression version with low Mdm4 expression. We found that the expression behavior of MDM4 is very similar to MDM2. Specifically, while MDM4 expression significantly decreased from early stage 1 in chRCC in comparison with normal tissues (Supplemental Figure S3AB), MDM4 expression significantly increased from early stage 1 in ccRCC (Supplemental Figure S3AC). Additionally, MDM4 expression increased in pRCC but had no significance (Supplemental Figure S3AD). Nevertheless, our TCGA data analysis indicated that the modulation of MDM4 expression in RCC versus in normal tissue has no effect on patient survival (Supplemental Figure S4). However, this may also not necessarily mean that MDM4 is not a target for tumor growth inhibition. As demonstrated in the case of HIF2α as a good target for ccRCC tumor elimination, while HIF2α high expression is associated with better survival in ccRCC.

## TP53/p53

TP53/p53 is the most studied tumor suppressor gene/protein. A PubMed search on March 21, 2021, showed that there are 40,097 publications containing p53 or TP53 in the publication title. In contrast, in the renal/kidney cancer area, the former criteria only resulted in 182 publications. This means that for the most relevant publications (i.e., key words in article titles), there is only one publication in the renal/kidney cancer area out of every 220 publications in PubMed. In this section, we will focus on the most relevant publications from the 182 publications on renal/kidney cancer. While many of these 182 publications investigated various situations/conditions that regulated different biological effects in renal cancer through p53 signaling such as these cited here [124–128], we will have a closer review of the p53/TP53’s role acting as a biomarker and/or therapeutic targets in renal/kidney cancer.

### p53 expression as a biomarker for RCC disease prognosis

**Fig. 14:**
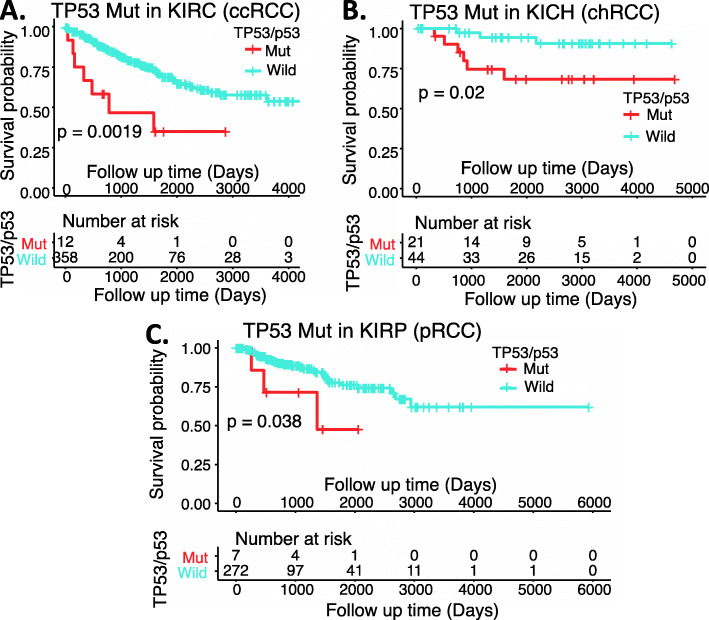
Effects of TP53/p53 mutation in RCC on patient survival probability: Kaplan-Meier survival analyses of the OS from TCGA-RCC cohorts of ccRCC (**A**), chRCC (**B**) and pRCC (**C**) were presented. Patients were grouped based on TP53/p53 mutation status. The log-rank test was used to determine the difference of OS for patients with TP53/p53 somatic mutation (red) versus without somatic mutation (blue). The figure was performed using R version 4.0.3

After review of the publications in the literature relevant to the use of p53 expression as a biomarker for RCC disease prognosis, it is unclear whether the detected p53 in RCC tissue was wild type or mutant. However, it is well known that wild type p53 usually has a very low-level expression or undetectable in cancer (due to MDM2-mediated binding, ubiquitination, and degradation of wild type p53 but not mutant p53). Secondly, in many (if not all) cases, the RCC patient cohort used for the studies/analyses of p53 expression in RCC tissues was not considered into the RCC major classifications. As mentioned earlier, RCC can be classified into 3 major histological subtypes: ccRCC/KIRC (70–80%), pRCC/KIRP (15–20%) (1), and chRCC/KICH (5–10%) (2) plus a minor subtype of cdRCC (< 1%). In consideration of these factors, we performed a somatic p53 mutation analysis in the 3 major types of RCC. Somatic mutations data were downloaded from Broad Firehose (http://firebrowse.org/) and TCGA MC3 Project [129]. Mutation Annotation Format (MAF) files were analyzed and visualized using the R Bioconductor package, maftools [130]. As shown in Supplemental Table S2, TP53/p53 in KICH/chRCC has a much higher mutation rate at 31.8% (21/66), while TP53/p53 in KIRC/ccRCC and KIR/pRCC has a much lower mutation rate at 3.24% (12/370) and 2.48% (7/282), respectively. Patient survival probability analysis of the individual RCC patient cohorts indicated that the TP53/p53 mutation is clearly associated with poor patient survival in all three major RCC types (Fig. 14), even though only a low-level somatic mutation of TP53/p53 in KIRC/ccRCC and KIRP/pRCC (Supplemental Table S2). Nevertheless, there was a wide variation in the reported incidence of p53 mutation in RCC. This is likely resulted from the cohort used in various studies with a high variation of the percentage among the three major RCC types as one major factor. By keeping the above information in mind, we have selected key representative publications to present the situation.

Uhlman et al. found that p53 mutation presented in 49 (28%) of 175 renal tumors, and p53 staining was associated with high tumor grade and stage [131]. Specifically, Authors used the p53 D07 (DO7/DO-7) monoclonal antibody (mAb, which recognizes all types of mutant p53 [132]) to immunohistochemically determine the p53 expression in paraffin-embedded nephrectomy specimens from 175 RCC patients [131]. Eleven (85%) of 13 metastatic lesions stained positively for p53, versus only four (36%) of the 11 paired primary tumors. Immunostaining for p53 was strongly associated with poor survival among patients without distant metastases [131]. In this group, 10-year disease-specific survival was 78% for patients with non-staining tumors versus 48% for those with p53-positive tumors (*p* ≤ 0.003). There was an 87% 10-year disease-specific survival rate for patients with non-staining Robson stage 1 tumors versus a 62% 10-year survival rate for patients with p53-positive Robson stage 1 tumors (*p* < 0.01). Multivariate analysis showed p53 immunoreactivity to be an independent predictor of survival for patients with nonmetastatic RCC but not tumor grade [131]. These authors proposed that (1) positive p53 immunostaining in RCC is associated with metastatic disease and poor survival in patients with early-stage disease, and (2) TP53/p53 mutations in RCC may contribute to the acquisition of metastatic potential [131]. Similarly, by using the D07/DO7/DO-7 p53 mAb Shvarts et al. immunohistochemically analyzed a tissue microarray from 366 patients with metastatic or localized RCC (193 localized RCC undergoing nephrectomy) for staining of CA9, CA12, Ki67, gelsolin, p53, EpCAM, pTEN and vimentin. These authors concluded that p53 is a significant molecular predictor of tumor recurrence in patients undergoing treatment for localized RCC [133]. By using DO-7 antibody, Mock et al. previously obtained similar results in RCC [134].

Interestingly, Noon et al. reviewed all of the studies that described the assessment of p53 and/or MDM2 in RCC and concluded that increased p53 expression, but not p53 mutation, is associated with reduced overall survival and more rapid disease progression in RCC [135]. However, in our view the question is whether the increased p53 expression in RCC tissues is actually mutant p53. One example cited by Noon et al. to support their conclusion is the paper from Lu et al. [136]. However, Lu et al. used the PAb1801 p53 mAb, which can recognize both wild type and mutant p53. Nevertheless, Lu et al. in their study clearly demonstrated that in bladder cancer, MDM2-positive tumors were highly associated with mutant p53 overexpression (Lu et al.’s Figs. 3, 4), which is associated with the high-risk, worse clinical prognosis category [136]. Lu et al. observed that MDM2-positive phenotype was significantly associated with early tumor stages in patients with poor survival [136]. Additionally, Noon et al. mentioned that the study from Warburton et al. supports the retention of relatively high levels of both wild-type p53 and MDM2 [137]. However, after inspecting this paper, we were unable to get this notion; instead, this paper only suggests that the wild type p53 is expressed and active in RCC cells and there was no convincing information related to p53 expression level [137]. Noon et al. also cited the paper from Haitel et al. [138] as an example that wild type p53 could be highly expressed in ccRCC. However, Haitel et al. used the p53 pan-antibody DO-1/DO1 for detecting p53 expression [138]; and this pan-antibody could recognize both wild type p53 and mutant p53 [132]. In our view, the Fig. 5 data from Haitel et al. [138] is likely that in the case of MDM2−/p53- or MDM2−/p53+ (associated with better patient survival), p53 should be wild type, but was only detected in some (but not all) of the paraffin-embedded specimens by using the p53 pan-antibody DO-1/DO1. This is because wild type p53 is usually expressed in a very low level and can be undetectable. Contrastingly, in the case of MDM2+/p53+ (associated worse patient survival), p53 should be mutant. In our view, given the DO1 mAb used that can detect both wild type p53 and mutant p53 [132], it would be unimaginable that RCC cells with high MDM2 expression could maintain a high wild type 53 expression. This is because it is well known that in cancer cells, MDM2 constitutively binds to, ubiquitinates and degrades wild type p53 via proteasome degradation pathway. So wild type p53 always maintains a low-level expression in cancer cells.

Nevertheless, after reviewing relevant publications, Noon et al. presented a conclusive summary: (1) MDM2 upregulation is associated with decreased disease-specific survival; (2) increased p53 expression is tightly linked with increased MDM2 expression; and, (3) patients who have tumors that display increased p53 and MDM2 expression may have the poorest overall survival [135]. Meanwhile, Noon et al. also stated that because there was no evidence to support the conclusion that p53 mutation is associated with poorer survival, it seemed clear that increased p53 expression in RCC occurs independent of mutation [135]. However, after we read relevant publications together with the Noon et al. review article, we reserve our notion that in most (if not all) cases, mutant p53 but not wild type p53 is overexpressed in MDM2-positive tumors in RCC and most likely in other cancer types as well. This final notion is based on our careful review of all relevant publications presented above.

Interestingly, one publication from Chemeris et al. reported that “Elevated content of p53 protein in the absence of p53 gene mutations as a possible prognostic marker for human renal cell tumors” [139]. Unfortunately, we are unable to access the full paper for this study. However, from the abstract, this study used DO-1 (which recognize both wild type p53 and mutant p53 [132]) for immunohistochemically determining p53 expression and used Pab240 to determine mutant p53 [139]. However, Pab240 is not sufficient in specificity [132] and in the denatured condition (e.g., in the Western blot and/or IHC methods) can recognize both wild type and mutant p53 (https://www.thermofisher.com/antibody/product/p53-Antibody-clone-PAb-240-Monoclonal/AHO0112). Therefore, the statement in the abstract that “Additional immunostaining of the positive samples with mutant p53-specific Pab240 mAb failed to detect immuno-positive material” is abnormal, since it should be stained regardless of the p53 being in a wild type or mutant status if using the p53 positive samples. Thus, such information plus only a limited area (exons 4–8) of p53 gene mutation being tested by the single strand conformation polymorphism (SSCP) analysis in their study blocked us to obtain a clear conclusion for this case.

More recently, Wang et al. performed a meta-analysis based on 22 studies including a total of 2013 RCC patients identified from various databases including PubMed [140]. The results showed that p53 positive expression is associated with poor overall survival (OS) (HR = 2.17, 95% confidence [CI]: 1.51–3.13) and cancer-specific survival (CSS) (HR = 1.59, 95% CI: 1.19–2.12) in RCC [140]. In addition, p53 positive expression was closely correlated with TNM stage (III/IV vs. I/II: OR = 2.51, 95% CI: 1.05–6.00), Fuhrman grade (III/IV vs. I/II: OR = 1.80, 95% CI: 1.24–2.63), and distant metastasis (M1 vs. M0: OR = 1.70, 95% CI: 1.16–2.49), but not related to lymph node involvement (N1 vs. N0: OR = 1.32, 95% CI: 0.80–2.18), primary tumor stage (pT3/pT4 vs. pT1/pT2: OR = 1.16, 95% CI: 0.88–1.53), and sex (*n* = 2, male vs. female, OR = 1.09, 95% CI: 0.70–1.68) [140]. These authors stated that given that the primary antibodies used for detecting p53 expression were inconsistent in different studies, this may contribute to heterogeneity [140]. These authors further stated in their paper that this study suggests that p53 positive expression is correlated with poor prognosis and advanced clinicopathological features in patients with RCC, which indicates that p53 is a potentially effective therapeutic target [140]. Similar results from more recent studies were also obtained by using the p53 DO-7 mAb [141], which recognizes all different mutant p53 [132].

Taken together, and based on our extensive review of the literature, we believe that the mentioned p53 positive expression likely means mutant p53 positive expression in most (if not all) cases. This notion is also consistent with the survival probability data that we presented in Fig. 14.

### Cancer therapeutics with p53: the story from CBLC137 (CBL0137)

Given that wild type p53 is a tumor suppressor, the most practical method is to use a strategy to reactivate p53 and/or increase its expression in RCC cells for RCC therapeutics. In this regard, since in most cases, RCC has wild type p53 (Supplemental Table S2), Gurova et al. proposed that p53 signaling in RCC might be repressed by some other mechanism. They found that all four RCC-derived cell lines (RCC26b, RCC45, RCC54, and RCC72) tested maintained wild-type p53. However, these cell lines were not capable of transactivating p53-responsive reporters and endogenous p53-responsive genes, although the p53 protein in RCC showed normal response to genotoxic stress (e.g., nuclear translocation, activation of specific DNA binding) [142]. These authors also found there are no indications of MDM2, MDM4, or ARF involvement in the functional repression of p53 in RCC; instead, p53-mediated transactivation can be activated by lentivirus vector-driven high-level expression of p53; and p53 inactivation prevailed in the hybrids of RCC cells with the cells possessing fully functional p53 [142]. The authors therefore proposed that a dominant inhibitor/mechanism is involved in p53-dependent transactivation repression in RCC [142]. In order to unravel the potential mechanism, these authors screened a diverse chemical compound library to search for small molecules that can restore p53-dependent transactivation of a p53-responsive reporter in RCC cells. They identified derivatives of 9-aminoacridine (9AA, Fig. 9G), including the antimalaria drug quinacrine (QC, Fig. 9H), which strongly induced p53 function in RCC as well as in other types of cancer cells [143]. They found that the induction of p53 by these compounds does not involve genotoxic stress but involves the suppression of NF-κB activity [143]. In contrast to agents that target IκB kinase 2, 9AA and QC can effectively suppress both basal and inducible activities of NF-κB, representing inhibitors of a previously undescribed type that convert NF-κB from a transactivator into a transrepressor [143]. Based on these findings, these authors proposed that the complete or partial repression of p53 observed in RCC and other cancer cells can be the result of constitutive activation of NF-κB [143]. In their view, the results from their study provide a possibility to kill cancer cells selectively through both inhibition of NF-κB and activation of p53 by a single small molecule [143].

Subsequently, these authors isolated and structurally optimized small molecules, curaxins (e.g., CBL0137, Fig. 9I), that simultaneously inhibit NF-κB and activate p53 without causing detectable genotoxicity [144]. Curaxins demonstrated anticancer activity against RCC and other human tumor xenografts tested in mice. Interestingly, these authors found that the effects of curaxins on p53, NF-κB, and their toxicity to cancer cells result from “chromatin trapping” of the histone chaperone FACT (facilitates chromatin transcription) complex; and the FACT inaccessibility leads to phosphorylation of the p53 Ser(392) by casein kinase 2 and inhibition of NF-κB-dependent transcription, which requires FACT activity at the elongation stage [144]. These results demonstrated that curaxins such as CBLC137 (CBL0137)-mediated inhibition of NF-κB and activation of p53 involve the FACT protein complex, which could be a new drug target.

Since then, the research rapidly expanded into other human cancers in the preclinical studies for the small molecule drug CBLC137 (CBL0137). This includes human pancreatic cancer [145]; neuroblastoma [146]; glioblastoma [147–149]; extremity melanomas [150]; small cell lung cancer [151, 152]; hepatocellular carcinoma [153]; leukemia [154]; and medulloblastoma [155]. Importantly, a phase 1 clinical trial result of CBL0137 from cancer patients with solid tumors was communicated at the ASCO 2020 Annual meeting [156]. This phase 1 is a dose-ranging study that assessed the CBL0137 maximum tolerated dose (MTD), recommended Phase 2 dose (RP2D), safety, pharmacokinetics (PK), and preliminary efficacy in adults with advanced treatment-refractory solid tumors. CBL0137 was administered via IV on Days 1, 8, and 15 of repeated 28-day cycles until progressive disease (PD) or unacceptable toxicity. Doses were escalated using a 3 + 3 design based on Cycle 1 dose-limiting toxicities (DLTs). PK was assessed through 168 h after Day 1. Efficacy was evaluated every 8 weeks. The study enrolled 83 patients (pts) (M/F [n] = 49/34; median [range] age = 64 [33–85] years; ECOG status [n] = 1/2 [32/51]), with cancer types (n) of colorectal (23 pts), prostate (7 pts), glioblastoma (6 pts), liver (6 pts), non-small-cell (5 pts), and others (36 pts) across 17 dose levels from 10 to 700 mg/m2/infusion. Durations of therapy ranged to 24 months. Cycle 1 DLTs (n type) were observed at 240 mg/m2 (1 Gr 3 photosensitivity), 400 mg/m2 (1 Gr 3 anemia), 700 mg/m2 (1 Gr 4 thrombocytopenia, 1 Gr 4 neutropenia/Gr 4 thrombocytopenia), and 650 mg/m2 (1 Gr 3 thrombocytopenia, 1 Gr 4 neutropenia/Gr 3 thrombocytopenia). Nausea and vomiting were successfully prevented with dexamethasone/serotonin antagonists. Photosensitization was effectively managed with sun protection. Peripheral venous thrombosis required central vein infusion in subjects with glioblastoma. PK showed dose-proportional increases in plasma CBL0137 area under the concentration-time curve (AUC), a high mean (range) volume of distribution (Vd) of 1030 (655–1460) L/m2 consistent with extensive tissue distribution and DNA intercalation, and an average mean (range) half-life (t1/2) of 24.7 (10.3–40.7) hours without dose dependence. The best response was stable disease: 2 patients with liver cancer had tumor control for 9 and 24 months and a maximum tumor regression of 10%; 2 patients with prostate cancer had tumor regressions by 11 and 22%; 1 patient with uterine cancer had a 20% tumor regression. These authors concluded that CBL0137 administered via IV was generally well tolerated with manageable toxicities and predictable PK, and the MTD and RP2D were estimated at 540 mg/m2 due to myelosuppressive DLTs. Preliminary evidence of antitumor activity supports Phase 2 testing [156].

Based on the results from the xenograft models, Lock et al. stated that the most consistent in vivo activity for CBL0137 was observed against acute lymphocytic leukemia (ALL) xenografts, with some solid tumor xenograft lines showing tumor growth delay [157]. In this regard, the next clinical trial for CBL0137 may focus on liquid/blood cancer instead of solid tumors, and this may be able to obtain a better preliminary efficacy for CBL0137.

### Cancer therapeutics with p53: the story from streptonigrin

Kim S-Y’s group showed that transglutaminase 2 (TGase2), a protein cross-linking enzyme, is markedly increased in RCC cell lines, and downregulation of TGase2 resulted in stabilizing p53 in parallel with the induction of 3–10-fold increase in apoptosis for RCC cell lines [158]. TGase2 directly cross links the DNA binding domain of p53, leading to p53 depletion in RCC via autophagy [158]. They also discovered that TGase2’s 1–139 residues interact with the MDM2-binding transactivation domain of p53’s 15–25 residues to compete with MDM2 for binding to p53 [159]. However, due to the use of different mechanisms for the TGase2-mediated degradation of p53 (p53 into autophagosome) versus MDM2-mediated degradation of p53 (via p53 ubiquitination), depletion of either of TGase2 or MDM2 induced p53 stabilization in RCC cells [159]. Next, this group screened a chemical library and identified streptonigrin (Fig. 9J) as a TGase2 inhibitor for potential RCC therapeutics [160]. They demonstrated the binding of streptonigrin to the N-terminus of TGase2 and importantly, a single dose of streptonigrin (0.2 mg/kg, daily × 5 per week, orally) showed marked antitumor effects associated with p53 stabilization in a preclinical RCC tumor model [160]. In our opinion, based on the data shown in the study, streptonigrin as a prototype TGase2 inhibitor exhibited a promising antitumor efficacy. However, since the toxicity of streptonigrin was not reported in the study, in order to move streptonigrin (or a streptonigrin analogue) into clinical trials, it would be important for a further study of the potential toxicity of streptonigrin (or a streptonigrin analogue).

## KRAS and AKT in RCC

KRAS has a high mutation rate in many cancer types including colorectal and pancreatic cancers. Mutant KRAS plays a critical role in treatment resistance. However, KRAS mutation is a rare event in RCC. KRAS mutation analysis of 121 RCCs of low-grade (*n* = 50) and advanced/metastatic (*n* = 71) subtypes indicated that only one sample has *KRAS*^G12D^ mutation [161]. Similarly, detection of the KRAS codon 12 mutation in 50 RCC tumors found no mutation [162]. The updated information from this study is roughly consistent with the three major types of RCC for KRAS mutation in RCC (Supplemental Table S2). However, while mutation of KRAS is a rare event in RCC, wild type KRAS may play a role in RCC cell proliferation and tumor growth. For example, tumor suppressor miRNA may exert their tumor suppressor effects by inhibiting KRAS signaling in RCC [163, 164]. Interestingly, as of March 21, 2021 there were only 46 publications containing the key words of “KRAS” or “K-RAS”, either in title or in abstract among the > 42,000 publications with renal/kidney cancer-related key words in the title. This might indicate that studying the role of KRAS in RCC is an insufficiently investigated area, which may provide an opportunity for researchers in the coming years.

Interestingly, while KRAS in RCC is understudied to date, AKT is a much more popular study area (18 publications with KRAS or K-ras in Title versus 168 publications with AKT in Title from over 42,000 kidney cancer publications as of March 21, 2021). Overview of these relevant publications indicated that many anti-RCC cell growth agents including various cellular proteins are through inhibiting the AKT signaling pathway to realize RCC cell growth/migration inhibition and apoptosis. We cited a few recent publications for an update [165–169]. In this regard, inhibition of AKT signaling pathway can be used for RCC cancer therapeutics.

Based on our collected kidney normal and RCC samples (Supplemental Table S1), we compared the expression of AKT1 (AKT), AKT2 and AKT3 in RCC tumor tissues versus normal tissues. We then divided the available RCC tumor samples into low versus high expression of AKT1, AKT2 and AKT3 and performed a patient survival association analysis. We now present these data below.

**Fig. 15:**
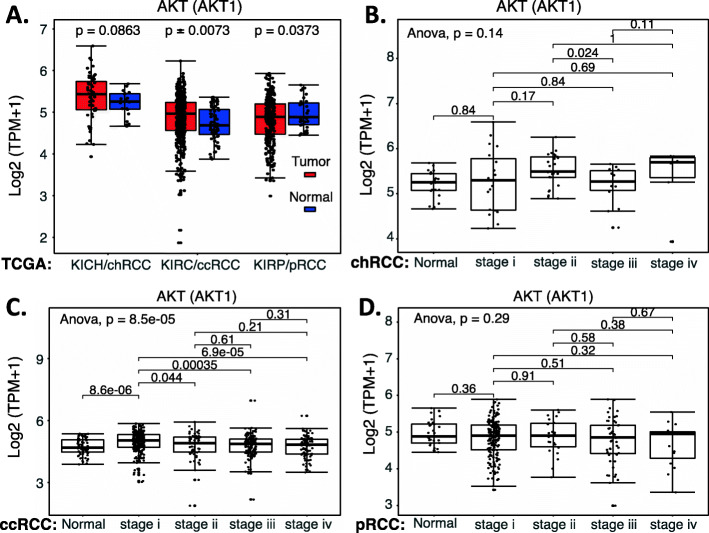
AKT1 expression in RCC tumor tissues versus in the associated normal tissue: Boxplots of the AKT1 expression level across TCGA-RCC subtypes in renal tumor (red) versus the associated normal renal tissues (blue) were presented (**A**). AKT1 expression among different stages of chRCC (**B**), ccRCC (**C**) and pRCC (**D**) versus the associated normal tissues was box-plotted. AKT1 expression was presented in the log2 (TPM + 1) scale format. Data were presented as the mean ± SD. A t-test was used to evaluate the statistical significance of the AKT1 mRNA expression level in normal renal tissues versus tumor tissues. One-way ANOVA was used to compare AKT1 expression among normal renal tissues versus different stages of RCC tumor tissues. The figure was performed using R version 4.0.3

**Fig. 16:**
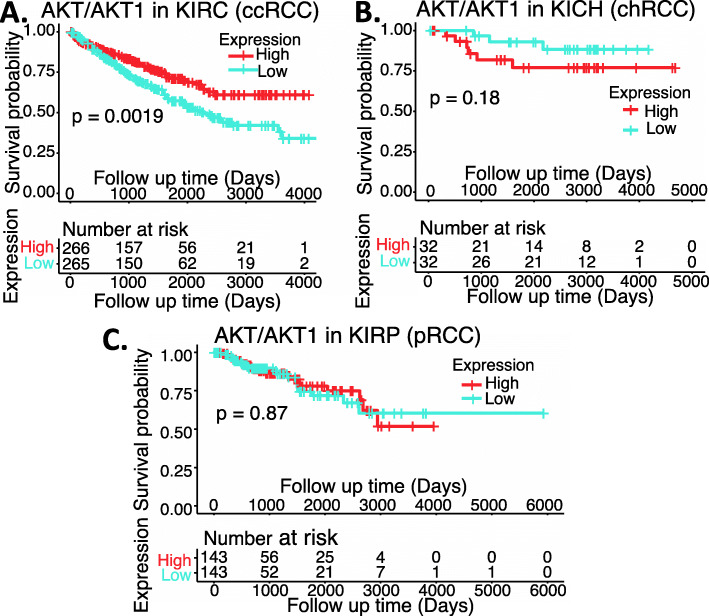
Effects of AKT1 expression on RCC patient survival probability: Kaplan-Meier survival analyses of OS from TCGA-RCC cohorts of ccRCC (**A**), chRCC (**B**) and pRCC (**C**) were presented. Patients were grouped into the high AKT1 expression group versus the low AKT1 expression group based on the median AKT1 mRNA expression. Each *p*-value for its significance from high versus low AKT1 expression was calculated using the log-rank test. The figures were performed using R version 4.0.3

In the case of AKT1, based on the outcome of these data, the expression of AKT1 is significantly increased in the early state 1 of ccRCC, while there are no significant AKT1 expression changes for the chRCC and pRCC (Fig. 15). Intriguingly, high expression of AKT1 in ccRCC is associated with better patient survival, while there is no significant association of AKT1 expression with patient survival for those with either chRCC or pRCC tumors (Fig. 16). Again, for many other cases discussed above, this may not necessarily mean that AKT is not a target for tumor growth inhibition. As in the case we mentioned early, while high expression HIF2α is associated with better patient survival in ccRCC, HIF2α was demonstrated to be a good target for ccRCC tumor elimination.

In the case of AKT2, while the expression of AKT2 is significantly decreased in the early stage 1 of chRCC and ccRCC (Supplemental Figure S5), none of the AKT2 low versus high expression in ccRCC, chRCC or pRCC is associated with a patient survival difference (Supplemental Figure S6).

**Fig. 17:**
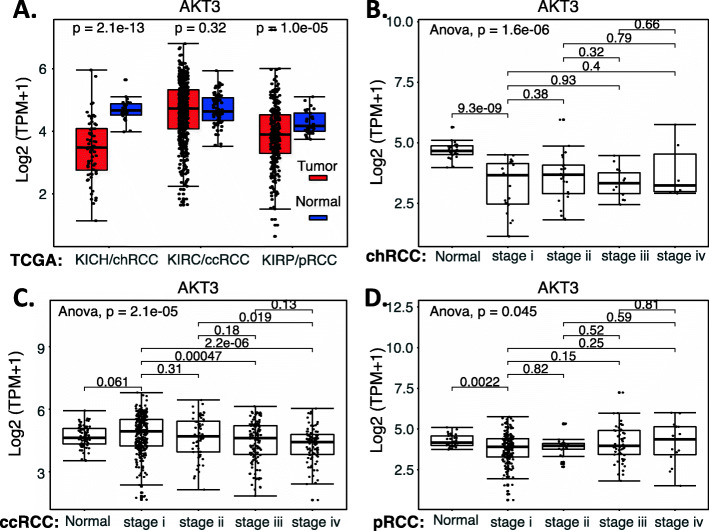
AKT3 expression in RCC tumor tissues versus in the associated normal tissue: Boxplots of the AKT3 expression level across TCGA-RCC subtypes in renal tumor (red) versus the associated normal renal tissues (blue) were presented (**A**). AKT3 expression among different stages of chRCC (**B**), ccRCC (**C**) and pRCC (**D**) versus the associated normal tissues was box-plotted. AKT3 expression was presented in the log2 (TPM + 1) scale format. Data were presented as the mean ± SD. A t-test was used to evaluate the statistical significance of the AKT3 mRNA expression level in normal renal tissues versus tumor tissues. One-way ANOVA was used to compare AKT3 expression among normal renal tissues versus different stages of RCC tumor tissues. The figure was performed using R version 4.0.3

**Fig. 18:**
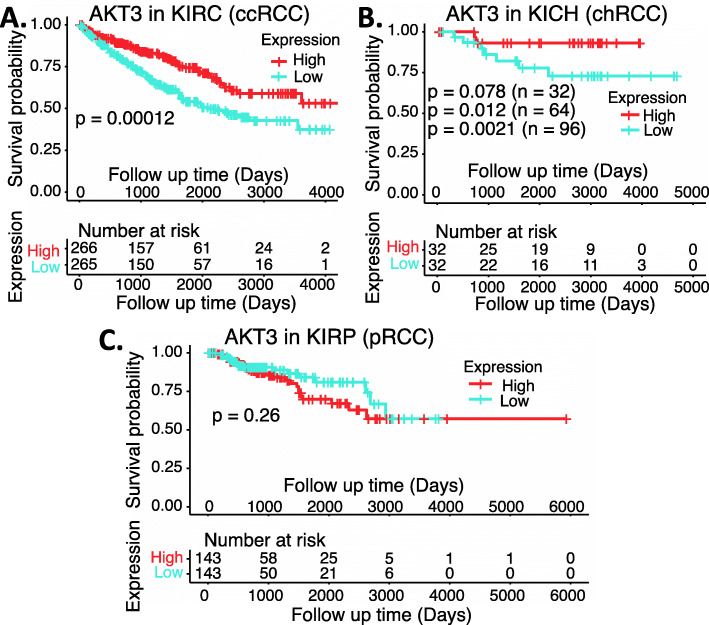
Effects of AKT3 expression on RCC patient survival probability: Kaplan-Meier survival analyses of OS from TCGA-RCC cohorts of ccRCC (**A**), chRCC (**B**) and pRCC (**C**) were presented. Patients were grouped into the high AKT3 expression group versus the low AKT3 expression group based on the median AKT3 mRNA expression. Each p-value for its significance from high versus low AKT3 expression was calculated using the log-rank test. The figures were performed using R version 4.0.3

In the case of AKT3, the expression of AKT3 in RCC tumor showed a huge variation in the individual RCC tumor samples. Generally, AKT3 expression is significantly decreased in the early stage 1 of chRCC and pRCC, while the overall AKT3 expression in ccRCC exhibited a wide variation change in comparison with the corresponding normal samples (Fig. 17). However, patient survival association indicated that high expression of AKT3 is associated with better ccRCC patient survival (Fig. 18A). For chRCC patients, high expression of AKT3 appears to also be associated with better patient survival (Fig. 18B), although due to the small cohort size, the *p*-value is at a margin. This notion is based on the fact that if we double or triple the cohort size from 32 to 64 or 96, the p-value would show high significance (Fig. 18B). However, for pRCC patients, it seems that high expression of AKT3 may be associated with worse patient survival, but the data is not significant although there is a sufficient cohort size (Fig. 18C). Again, we should always keep in mind that TCGA mRNA data for gene expression may not always reflect the protein expression. Furthermore, this may not necessarily mean that AKT3 is not a target for tumor inhibition. As demonstrated for the case of HIF2α.

## Could the small molecule FL118, or an FL118 analogue be developed for RCC treatment?

The chemical name of FL118 is shown in Fig. 9K. We discovered FL118 by using cancer cell models that are genetically engineered with the survivin gene promoter-driven luciferase reporter as an assay system [170] via high throughput screening; followed by in vitro-&-in vivo hits-to-lead analyses [171]. Although the chemical structure of FL118 is similar to camptothecin (CPT) and its analogues, irinotecan, SN-38 (the active metabolite of irinotecan) and topotecan, FL118 has a unique structure of “10,11-methylenedioxy”; none of the other CPTs (Fig. 9KLMNO) has this structure. Consistent with its unique chemical structure, FL118 does not use topoisomerase I (Top1) as its therapeutic target. Specifically, inhibition of Top1 activity by FL118 is at the μM level, while inhibition of cancer cell growth by FL118 is in the range of pM (sub-nM) to nM levels [171]. While CPTs need the expression of Top1 targets for their cancer therapeutic effectiveness [172–174], FL118 can eliminate human tumors that have no Top1 expression [175]. Instead of using Top1 as its therapeutic target, FL118 inhibits multiple drug resistance proteins (survivin, MCL-1, XIAP, cIAP2, MDM4) [171, 176] and key DNA damage repair regulators, ERCC1 [177] and ERCC6 [178]. Furthermore, while ABC efflux pump protein transporters are known to be drug resistance factors for CPTs [179–183], FL118 is not a substrate of efflux pump protein transporters such as ABCG2/BCRP and MDR1/Pgp [184, 185], and can bypass such protein expression-mediated drug resistance [184, 185]. Additionally, TP53/p53 mutations or KRAS mutations are well known to be challenging treatment resistant factors. However, FL118 exhibited better anticancer efficacy in colorectal cancer (CRC) cells with mutant p53 [176], and our recent studies indicated that FL118 exhibited even better anticancer efficacy either in human bladder cancer cells with KRAS mutation or in human CRC cells with KRAS mutation in comparison with bladder cancer cells or CRC cells with wild type KRAS [186, 187].

Furthermore, cancer stem cells (CSC) are known to play a critical role in treatment resistance and metastasis. Accordingly, FL118 inhibits CSC markers/targets (ABCG2, ALDH1A1, Oct4) and reduces the invasive capability of CSC spreading [177]. Consistently, FL118 eliminates human pancreatic ductal adenocarcinoma (PDAC) tumors and inhibits human PDAC metastasis in animal models [178]. Importantly, FL118 is orally available, is highly stable chemically, accumulates and resides in tumors, and is rapidly cleared from the bloodstream (favorable pharmacokinetics - PK) [184]. The available data indicates that FL118 exhibits favorable toxicity profiles in mice and dogs [178]. Finally, recent studies from our collaborators demonstrated that the bone marrow stromal cells-rendered multiple myeloma (MM) cell resistance to CAR T cells can be abrogated by FL118 treatment through the inhibition of survivin, MCL-1 and XIAP [188].

These FL118 features prompted us to evaluate the FL118 therapeutic potential and the potential modulation of various protein targets by FL118 in RCC. Results derived from our studies are presented below.

**Fig. 19:**
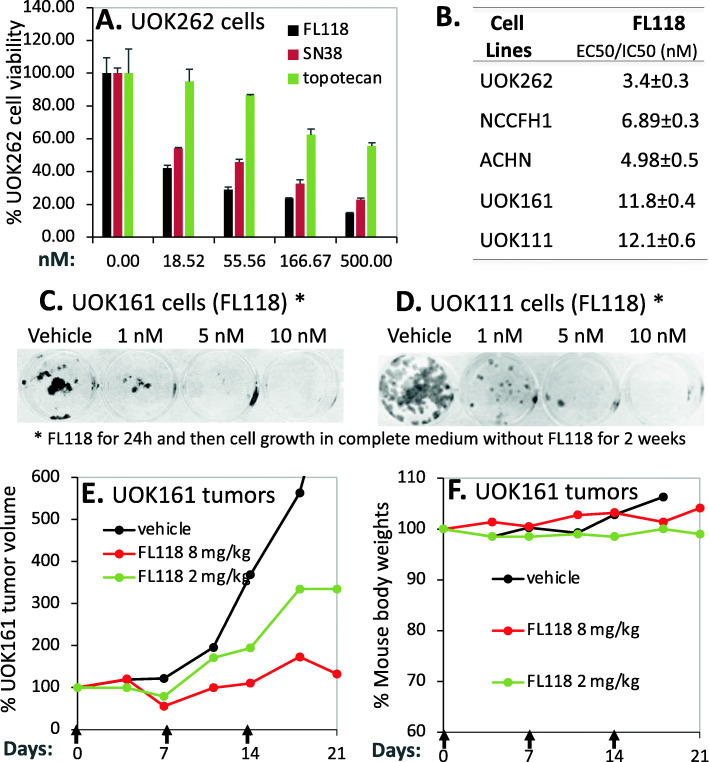
FL118 in vitro (ex vivo) and in vivo efficacy for RCC: **A** FL118 inhibition of the FHpRCC cell growth. FHpRCC UOK262 cells in 96-well plates were treated with and without FL118, SN-38 and topotecan for 72 h with a series of drug concentrations as shown. Cell viability was then determined using MTT assay. The data is the mean ± SD from three independent assays. **B** IC_50_ values derived from two FHpRCC Type 2 cell lines (UOK262, NCCFH1), one Type 1/2 pRCC cell line (ACHN) and two ccRCC cell lines (UOK161, UOK111) are shown. IC50 were calculated from cell growth inhibition determined using MTT assay. **C**, **D** 50–100 RCC cells were seeded in a total of 2 ml complete media in each well of 12 Well-plates and allowed to attach for 24 h prior to treatment. Cells were then treated with vehicle or FL118 (1, 5, 10 nM) for 24 before changing the complete media without FL118. Cells were then allowed to grow for 2 weeks in the incubator with complete media changed every 72 h. when the colonies were of sufficient size (> 50 cells), media was aspirated, and wells containing colonies were washed with 1X PBS and then fixed using 1 ml of 100% methanol for 5–10 min. Methanol was then aspirated, and colonies were stained using 1 ml of 0.5% Crystal violet for 20 min. Plates were then washed with tap water until cleared and dried before images were taken. **E** Inhibitory effects of FL118 on UOK161 cell-established xenograft tumor growth. Xenograft tumor establishment: UOK161cells grown in complete medium were harvested and 5-million cells mixed with 50% matrigel per site were injected in the flank area of SCID (severe combined immunodeficiency) mice. After the tumors reached 100–200 mm^3^ (designated as day 0), vehicle or FL118 were orally administrated (arrowed) with two doses (2 mg/kg, 8 mg/kg) on days 0, 7 and 14. Of note, FL118 MTD is 10 mg//kg with the weekly schedule. **F** Mouse body weight changes

We first used the FHpRCC cell line UOK262 cells to compare the drug-mediated cell growth inhibition measured by MTT assay for FL118, SN-38 (irinotecan’s active metabolite) and topotecan. We found that FL118 exhibits a significantly better capability to inhibit UOK262 cell growth in comparison with SN38 or topotecan (Fig. 19A). We then determined FL118-mediated growth inhibition using MTT assay for other RCC cell lines including two FHpRCC cell lines (UOK262, NCCFH1), one Type 1/2 pRCC cell line (ACHN) and two ccRCC cell lines (UOK161, UOK111). We then analyzed the FL118 IC_50_ for the inhibition of cell growth/viability. The results are presented in Fig. 19B. It appears that UOK161 and UOK111 have IC_50_ higher than other RCC cell lines. We therefore performed colony formation assay using UOK161 and UOK111 cells and found that FL118 exhibited a high ability to inhibit UOK161 and UOK111 cell colony formation (Fig. 19CD) and 10 nM FL118 for 24 h treatment could completely eliminate UOK161 and UOK111 cell colony formation (Fig. 19CD). Next, we established UOK161 xenograft tumors in SCID mice and performed antitumor activity of FL118 in two sub-MTD doses (2 mg/kg, 8 mg/kg). We found that FL118 exhibited significant antitumor activity in both dose levels (Fig. 19E), while showing no clinical observations of toxicity including mouse body weight changes (Fig. 19F).

**Fig. 20:**
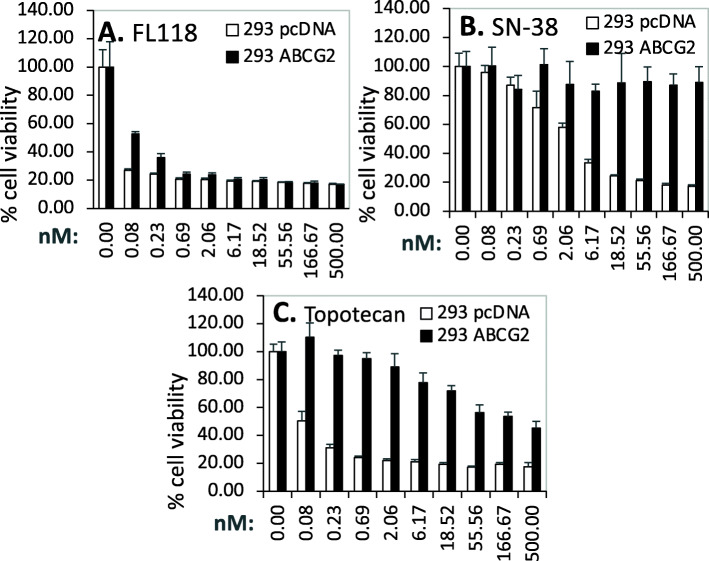
Differential sensitivity of ABCG2-overexpressed HEK293 cells to FL118, SN-38 and topotecan: Sub-confluent HEK293 cells that have been stably transfected with either pcDNA3 empty vectors (control) or pcDNA3-ABCG2 expression vectors were treated with or without a series of concentrations of FL118 (**A**), SN-38 (**B**) and topotecan (**C**) for 72 h as shown. Cell viability was then determined using MTT assay. Each bar is the mean cell growth ± SD derived from 5 independent parallel testing

ABC transporter efflux pump proteins are known to be important drug treatment-resistant factors and the ABCG2/BCRP efflux pump protein is a critical one, which is involved in cancer stem cells’ survival and function. We therefore used human embryo kidney (HEK) 293 cells that were transfected with pcDNA3 empty vectors versus with pcDNA3-ABCG2 expression vectors to alternatively compare the differential sensitivity of these genetically modified model cells with FL118, SN-38 and topotecan. Consistent with our previous finding [185], we found that while overexpression of ABCG2 significantly increases the SN-38 and topotecan resistance (Fig. 20BC), overexpression of ABCG2 in HEK293 cells does not increase resistance to FL118 (Fig. 20A), indicating that FL118 is not a substrate of the efflux pump protein ABCG2. These findings are consistent with our previous findings in other cancer cell types (e.g., CRC) [185].

**Fig. 21:**
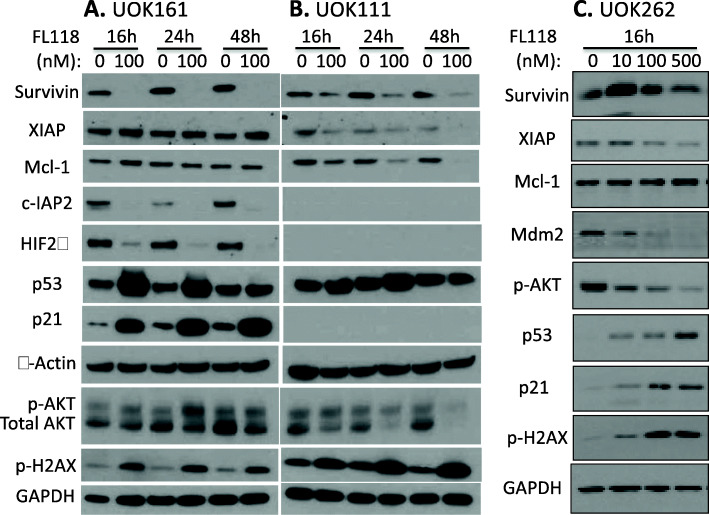
Effects of FL118 on the modulation of potential molecular targets in the ccRCC cell lines UOK161, UOK111) and the Type 2 pRCC cell line, FHpRCC UOK262: Sub-confluent cells grown in complete medium in 6-well plates were treated with and without FL118 at the concentration of 100 nM for 16 h, 24 h and 48 h (**A**, **B**) or for 16 h at the concentration of 10 nM, 100 nM and 500 nM (**C**) as shown. Cells were then lysed for Western blot analyses with antibodies for the corresponding molecular protein targets. The expression of β-actin and GAPDH are the internal protein loading control as shown. Of note, HIF1α, MDM2 and Mdm4 were not detected in both UOK161 and UOK111 cells

We then determined a set of relevant target proteins in RCC cells via Western blot analyses (Fig. 21). Specifically, in the ccRCC cell lines UOK161 and UOK111, FL118 could effectively inhibit the expression of survivin and HIF2α, and activate p53 pathway (p53, p21) in association with the induction of the double-stranded DNA break marker p-H2AX (Fig. 21AB), while the inhibitory effects of FL118 to XIAP is cell line-dependent (i.e., no inhibitory effects in UOK161 but moderate inhibited in UOK111). Intriguingly, FL118 inhibits both p-AKT and total AKT (AKT1) expression in both UOK161 and UOK111 cells. Additionally, FL118 could effectively inhibit cIAP2 expression in UOK161 cells (Fig. 21A). However, the significance of FL118 inhibition of cIAP2 contributing to FL118 anti-ccRCC tumor needs further investigation, because only one publication focused on the studies of cIAP1 and cIAP2 in RCC without a conclusive role for cIAP2 [189].

A similar molecular protein target modulation by FL118 in FHpRCC UOK262 cells is also obtained (Fig. 21C). Based on the data, the FL118 molecular protein targets are highly overlapped between ccRCC cells (UOK161, UOK111) and pRCC cells (UOK262). In addition to the modulation of survivin, HIF2 and p53, etc. by FL118, FL118 could inhibit both p-AKT and total AKT expression (Fig. 21). In this regard, both total AKT expression and phosphorylation are involved in RCC cell migration/invasion, metastasis, drug-induced cell death and RCC development [165–169]. Thus, AKT may also be used as a target for RCC [190, 191].

**Fig. 22:**
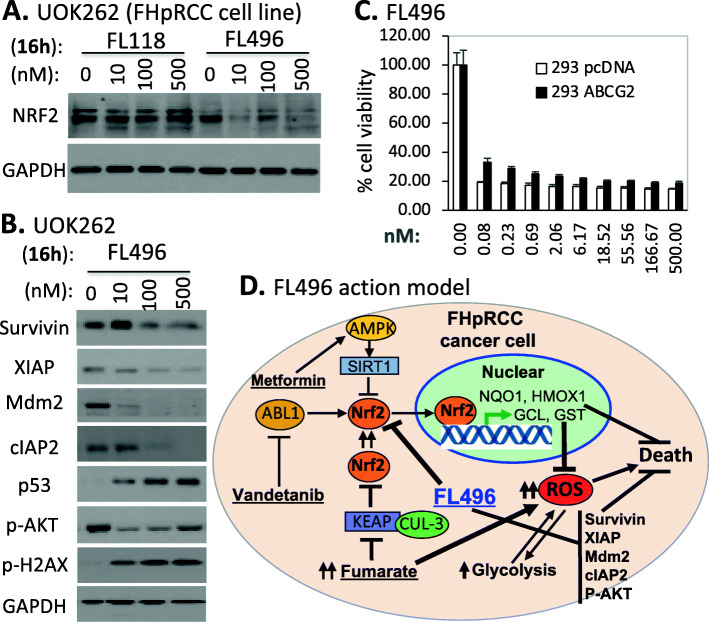
FL496, a FL118 analogue, exhibited extended target specificity: **A** Effects of FL118 and FL496 on the expression of NRF2 in UOK262 tumor cells. Sub-confluent cells grown in complete medium in 6-well plates were treated with FL118 and FL496 for 16 h at 0, 10, 100 and 500 nM, respectively as shown. Cells were then analyzed with Western blots using the NRF2 antibody. GAPDH is the internal protein loading control. **B** Effects of FL496 on the modulation of potential molecular targets in the Type 2 pRCC cell line, FHpRCC UOK262. Sub-confluent cells grown in complete medium in 6-well plates were treated for 16 h with FL496 at the concentration of 0 nM, 10 nM, 100 nM and 500 nM as shown. Cells were then lysed for Western blot analyses with antibodies for the corresponding molecular protein targets. GAPDH is the internal protein loading control. **C** FL496 is not an ABCG2 efflux pump substrate: HEK293 cells stably transfected with either pcDNA3 empty vectors (control) or with pcDNA3-ABCG2 expression vectors were treated with or without a series of concentrations of FL496 for 72 h as shown. Cell viability was then analyzed using MTT assay. Each bar is the mean cell growth/viability ± SD derived from 5 independent parallel testing. **D** FL496 mechanism of action model based on the data observed (**A**, **B**), which was adapted from the publication entitled “Targeting ABL1-Mediated Oxidative Stress Adaptation” [192].

Intriguingly, we have preliminary data showing that certain FL118 analogues could have extended target specificity and exhibit extraordinary anticancer activity in treating cancer cells with certain specialized genetic/epigenetic alterations in comparison with FL118 itself. For example, in terms of FHpRCC in which NRF2 is a critical target for the disease [1], FL496 (a FL118 Position 7 analogue) exhibited a capability to potentially inhibit NRF2 expression, but FL118 could not (Fig. 22A), while both FL118 and FL496 have highly overlapped molecular protein targets (compare Fig. 21C to Fig. 22B). Additionally, our data also showed that both FL118 (Fig. 20A) and FL496 (Fig. 22C) are not the ABCG2 substrate, while both SN-38 and topotecan are ABCG2 substrates (Fig. 20BC).

Based on these molecular targeting features of FL496 and the molecular pathway for FHpRCC tumors, we outlined a FL496 mechanism of action model (Fig. 22D). Previous studies indicated that the protooncogene product ABL1 can activate NRF2 and vandetanib could inhibit ABL1, and thus vandetanib exhibited effective anti-UOK262-established xenograft tumor [192]. Based on FL496’s inhibitory effects on anti-FHpRCC cell/tumor models, FL496 used for treating the specialized RCC cancer (FHpRCC) is warranted for further investigation.

Finally, several lines of evidence suggest that RCC is likely a unique type of cancer that is genetically and/or epigenetically different from other cancer types. First, in contrast to KRAS mutation in many cancer types including CRC and PDAC with a high mutation rate, KRAS mutation in RCC is a rare event (Supplemental Table S2). Thus, the significance for KRAS mutation in RCC is unclear. Second, our previous studies found that in CRC cells, FL118 treatment could induce MDM2 expression and switch the FL118-induced MDM2 from an oncogenic protein (i.e., ubiquitination and degradation of p53) to a tumor suppressor protein (i.e., ubiquitination and degradation of its oncogenic partner MDM4) [176]. However, in the FHpRCC UOK262 cells, FL118 inhibits but does not induce the expression of MDM2 (Fig. 21C).

## Conclusions

Survivin is overexpressed in RCC and is a promising oncogenic biomarker and target for RCC in addition to other cancer types. However, whether exclusively targeting survivin for RCC therapeutics is sufficient for RCC patients with survivin-overexpressed tumors is still a question that requires further investigation. Although limited studies support a role of XIAP and MCL-1 for treatment resistance in RCC, there is no strong data to support that XIAP and MCL-1 are good biomarkers for RCC prognosis. Additional studies would be required in order to draw a clear conclusion. There are inconsistent studies on HIF1α acting as RCC-favorable or unfavorable biomarker and target. Some studies showed HIF1α acting as an oncogenic role, while others showed HIF1α acting as a tumor suppressor role. It is likely that HIF1α plays both oncogenic roles (less common) and tumor suppressor roles (more common), which depends on the signal network context and/or cell type. Nevertheless, use of HIF1α as an oncogenic target for RCC therapeutics remains to be demonstrated. In contrast, while certain inconsistent studies on HIF2α exist, most studies demonstrated that HIF2α is a good oncogenic biomarker and target at least for ccRCC tumor therapeutics. Interestingly, our TCGA data (Fig. 8B) indicated that the highly expressed HIF2α mRNA in ccRCC is associated with better patient survival. Nevertheless, studies have demonstrated that small molecules targeting HIF2α exhibited good anti-ccRCC tumor activities. This suggests that high HIF2α is a treatment resistant factor, while high HIF2α in RCC before treatment may have a mechanism to stabilize the disease. While targeting NRF2 for cancer therapeutics is an active area overall in other cancer types, NRF2 is mainly involved in a subset of type 2 pRCC (i.e., FHpRCC) and NRF2 is an essential target for FHpRCC tumors. Some studies have shown that high expression of MDM2 is an aggressive RCC biomarker, which could be the result from the multiple roles of MDM2 in RCC signaling as suggested in some studies. However, our TCGA genetic data does not support MDM2 or MDM4 being good biomarkers for RCC. However, this may not necessarily mean that they could not be used as therapeutic targets. For example, as in the case of HIF2α, while HIF2α mRNA high expression linked to better ccRCC patient survival, studies still demonstrated that HIF2α is a good target for treatment of ccRCC. TP53/p53 mutation is a strong oncogenic biomarker and overexpression of mutant p53 is an unfavorable RCC biomarker. The wild type p53 function is suppressed in RCC cells in many situations. In this case, the reactivation of wild type p53 in RCC has cancer therapeutics effects. The small molecule CBL0137 demonstrated its antitumor activity not only in RCC but also in other cancer types, which is likely because the single small molecule CBL0137 could at the same time inhibit NF-κB and activate p53. KRAS mutation in RCC is a very rare event. However, the wild type KRAS activity may still play a role in RCC cell proliferation and tumor growth. Finally, increased AKT expression/activation in cancer cells results in growth advantage. However, our TCGA genetic data indicated that the high expression of AKT1 (AKT) and AKT3 mRNAs is associated with better prognosis in ccRCC. However, this observation may not rule out that AKT cannot be used as targets as in the case of HIF2α.

RCC has additional biomarkers and targets for potential therapeutics. We have reviewed a limited set of relevant biomarkers and targets (survivin/BIRC5, XIAP, MCL-1, HIF1α, HIF2α, NRF2, MDM2, MDM4, TP53/p53, KRAS, AKT) in this article. It is likely that an anti-RCC agent does not need to inhibit all the potential RCC biomarkers/targets for obtaining a high efficacy. However, inhibition of multiple therapeutic targets by one agent would likely have a better chance for obtaining higher anticancer efficacy. In this regard, given that FL118 appears to inhibit multiple therapeutic targets in RCC, it will be intriguing to see whether the small molecule FL118 or an analogue of FL118 (e.g., FL496 shown in Fig. 22) could go through the drug development process into clinical trials for treating certain types of RCC patients.

## Supplementary Information

**Additional file 1: Supplemental Material 1.** Role of NRF2 in non-cancerous disease prevention and aging retardation. **Supplemental Table S1.** Summary of the datasets collected from publicly available kidney tumor tissues versus normal tissue samples*. **Supplemental Table S2.** Somatic Mutation Analysis Summary of relevant genes*. **Supplemental Table S3.** Classification of KIRP/pRCC tumor and normal tissues into Type 1 pRCC and Type 2 pRCC. **Supplemental Figure S1.** NRF2 expression in Type 1 pRCC and Type 2 pRCC tumor tissues versus in normal renal tissues: Boxplots of the NRF2 expression level across TCGA Type 1 or Type 2 pRCC tumor tissues (red) versus the normal renal tissues (blue) were presented (**A**). NRF2 expression among different stages of Type 1 pRCC tumor tissues versus the matched normal tissue (**B**) or versus all normal tissues (**C**) was box-plotted. NRF2 expression among different stages of Type 2 pRCC tumor tissues versus matched normal tissue was box-plotted (**D**). NRF2 expression was presented in the log2 (TPM + 1) scale format. Data was presented as the mean ± standard deviation (SD). A t-test was used to evaluate the statistical significance of the NRF2 mRNA expression level in renal normal tissues versus either Type 1 or type 2 pRCC tumor tissues. One-way ANOVA was used to compare NRF2 expression among renal normal tissues versus different stages of Type 1 or Type 2 pRCC tumor tissues. The figure was performed using R version 4.0.3. **Supplemental Figure S2.** Effects of NRF2 expression on Type 1 pRCC patient survival probability: Kaplan-Meier survival analyses of overall survival (OS) from TCGA-Type 1 (**A**) or Type 2 (**B**) pRCC cohorts were presented. Patients were grouped into the high NRF2 expression group versus the low NRF2 expression group based on the median NRF2 mRNA expression level in either Type 1 (**A**) or Type 2 (**B**) pRCC tumor tissues. Each *p*-value for the significance from high versus low NRF2 expression was calculated using the log-rank test. The figures were performed using R version 4.0.3. **Supplemental Figure S3.** Mdm4/MdmX expression in RCC tumor tissues versus in normal tissues: Boxplots of the Mdm4MdmX expression level across TCGA-RCC subtypes in renal tumor (red) versus the associated normal renal tissues (blue) were presented (**A**). Mdm4MdmX expression among different stages of chRCC (**B**), ccRCC (**C**) and pRCC (**D**) versus normal renal tissues was box-plotted. Mdm4MdmX expression was presented in the log2 (TPM + 1) scale format. Data was presented as the mean ± SD. A t-test was used to evaluate the statistical significance of the mRNA expression level in renal normal versus tumor tissues. One-way ANOVA was used to compare Mdm4MdmX expression among renal normal tissues versus different stages of RCC tumor tissues. The figure was performed using R version 4.0.3. **Supplemental Figure S4.** Effects of Mdm4/MdmX expression on RCC patient survival probability: Kaplan-Meier survival analyses of OS from TCGA-RCC cohorts of ccRCC (**A**), chRCC (**B**) and pRCC(**C**) were presented. Patients were grouped into the high Mdm4/MdmX expression group versus the low Mdm4/MdmX expression group based on the median Mdm4/MdmX mRNA expression. Each p-value for the significance from high versus low Mdm4/MdmX expression was calculated using the log-rank test. The figures were performed using R version 4.0.3. **Supplemental Figure S5.** AKT2 expression in RCC tumor tissues versus in normal tissues: Boxplots of the AKT2 expression level across TCGA-RCC subtypes in renal tumor (red) versus the associated normal renal tissues (blue) were presented (**A**). AKT2 expression among different stages of chRCC (**B**), ccRCC (**C**) and pRCC (**D**) versus normal renal tissues was box-plotted. AKT2 expression was presented in the log2 (TPM + 1) scale format. Data was presented as the mean ± SD. A t-test was used to evaluate the statistical significance of the mRNA expression level in renal normal versus tumor tissues. One-way ANOVA was used to compare AKT2 expression among renal normal tissues versus different stages of RCC tumor tissues. The figure was performed using R version 4.0.3. **Supplemental Figure S6.** Effects of AKT2 expression on RCC patient survival probability: Kaplan-Meier survival analyses of OS from TCGA-RCC cohorts of ccRCC (**A**), chRCC (**B**) and pRCC (**C**) were presented. Patients were grouped into the high AKT2 expression group versus the low AKT2 expression group based on the median AKT2 mRNA expression. Each p-value for the significance from high versus low AKT2 expression was calculated using the log-rank test. The figures were performed using R version 4.0.3.

## Data Availability

Refer to the information provided in the Review article and in the supplemental information document.

## Abbreviations

BIRC5: Baculoviral IAP repeat-containing protein 5
CPT: Camptothecin
CTL: Cytotoxic T
HIF: Hypoxia inducible factor
IAP: Inhibitor of apoptosis protein
ICI: Immune checkpoint inhibitor
IHC: Immunohistochemistry
MCL-1: Myeloid cell leukemia-1
NRF2: Nuclear factor erythroid 2-related factor 2
RCC: Renal cell carcinoma
siRNA: Small interfering RNA
XIAP: X-linked Inhibitor of apoptosis
