# Supplementary material for: Kidney cancer biomarkers and targets for therapeutics: survivin (BIRC5), XIAP, MCL-1, HIF1α, HIF2α, NRF2, MDM2, MDM4, p53, KRAS and AKT in renal cell carcinoma
Source: J Exp Clin Cancer Res. 2021 Aug 12;40:254. doi: 10.1186/s13046-021-02026-1 (PMC8359575; doi:10.1186/s13046-021-02026-1)
Supplement: Supplementary file 1 — Additional file 1: Supplemental Material 1. Role of NRF2 in non-cancerous disease prevention and aging retardation. Supplemental Table S1. Summary of the datasets collected from publicly available kidney tumor tissues versus normal tissue samples*. Supplemental Table S2. Somatic Mutation Analysis Summary of relevant genes*. Supplemental Table S3. Classification of KIRP/pRCC tumor and normal tissues into Type 1 pRCC and Type 2 pRCC. Supplemental Figure S1. NRF2 expression in Type 1 pRCC and Type 2 pRCC tumor tissues versus in normal renal tissues: Boxplots of the NRF2 expression level across TCGA Type 1 or Type 2 pRCC tumor tissues (red) versus the normal renal tissues (blue) were presented (A). NRF2 expression among different stages of Type 1 pRCC tumor tissues versus the matched normal tissue (B) or versus all normal tissues (C) was box-plotted. NRF2 expression among different stages of Type 2 pRCC tumor tissues versus matched normal tissue was box-plotted (D). NRF2 expression was presented in the log2 (TPM + 1) scale format. Data was presented as the mean ± standard deviation (SD). A t-test was used to evaluate the statistical significance of the NRF2 mRNA expression level in renal normal tissues versus either Type 1 or type 2 pRCC tumor tissues. One-way ANOVA was used to compare NRF2 expression among renal normal tissues versus different stages of Type 1 or Type 2 pRCC tumor tissues. The figure was performed using R version 4.0.3. Supplemental Figure S2. Effects of NRF2 expression on Type 1 pRCC patient survival probability: Kaplan-Meier survival analyses of overall survival (OS) from TCGA-Type 1 (A) or Type 2 (B) pRCC cohorts were presented. Patients were grouped into the high NRF2 expression group versus the low NRF2 expression group based on the median NRF2 mRNA expression level in either Type 1 (A) or Type 2 (B) pRCC tumor tissues. Each p-value for the significance from high versus low NRF2 expression was calculated using the log-rank test. The figures were p [file 13046_2021_2026_MOESM1_ESM.docx]

**Supplemental Material 1:**

**Role of NRF2 in non-cancerous disease prevention and aging retardation**

NRF2 (Nuclear factor erythroid 2-related factor 2) is a transcription factor and can increase the production of several antioxidant enzymes that can eliminate reactive oxygen species (ROS). Therefore, NRF2 is a major player in decreasing ROS-induced oxidative stress that can harm normal tissues/cells. Studies on the development and use of NRF2 agonists/activators to decrease/eliminate ROS production for human health and delay aging have been an active research area for decades. For example, the potential application of NRF2 activators in intracranial hemorrhage (1); development and optimization of halogenated vinyl sulfones as NRF2 activators for treatment of Parkinson's disease (2); a hydrogen peroxide responsive prodrug of Keap1-NRF2 inhibitor for improving oral absorption and selective activation in inflammatory conditions (3) and the use of WJ-39, an aldose reductase inhibitor, to ameliorate renal lesions in diabetic nephropathy by activating NRF2 signaling (4). For clarification, the terms used in studies related to the Keap1-NRF2 (protein-protein) interaction inhibitors actually are NRF2 activators but not NRF2 inhibitors (5, 6). This is simply because Cul3-Keap1 binds to and degrades NRF2 protein (7, 8), and thus breaks their protein-protein interaction, NRF2 would be released and activated. Therefore, the Keap1-NRF2 (interaction) inhibitors should be called as Keap1-NRF2 interaction disruptors or Keap1 inhibitors (9), thus avoiding the potential confusion. Generation of various types of Keap1 inhibitors (i.e., NRF2 agonists/activators) for non-cancerous disease treatment is a growing and highly active research area in past decades. We anticipate that this area will continue and may provide promising therapeutics for non-cancerous diseases and/or sub-healthy conditions in the coming years. However, detailed review of this area is out of the coverage of this review. For more information, readers may refer to recent review articles(10, 11).

**References:**

1. Imai T, Matsubara H, Hara H. Potential therapeutic effects of Nrf2 activators on intracranial hemorrhage. J Cereb Blood Flow Metab. 2021:271678X20984565.

2. Choi JW, Kim S, Yoo JS, Kim HJ, Kim HJ, Kim BE, et al. Development and optimization of halogenated vinyl sulfones as Nrf2 activators for the treatment of Parkinson's disease. Eur J Med Chem. 2021;212:113103.

3. Lu M, Zhang X, Zhao J, You Q, Jiang Z. A hydrogen peroxide responsive prodrug of Keap1-Nrf2 inhibitor for improving oral absorption and selective activation in inflammatory conditions. Redox Biol. 2020;34:101565.

4. Zhou X, Liu Z, Ying K, Wang H, Liu P, Ji X, et al. WJ-39, an Aldose Reductase Inhibitor, Ameliorates Renal Lesions in Diabetic Nephropathy by Activating Nrf2 Signaling. Oxid Med Cell Longev. 2020;2020:7950457.

5. Zhou HS, Hu LB, Zhang H, Shan WX, Wang Y, Li X, et al. Design, Synthesis, and Structure-Activity Relationships of Indoline-Based Kelch-like ECH-Associated Protein 1-Nuclear Factor (Erythroid-Derived 2)-Like 2 (Keap1-Nrf2) Protein-Protein Interaction Inhibitors. J Med Chem. 2020;63(19):11149-68.

6. Lazzara PR, David BP, Ankireddy A, Richardson BG, Dye K, Ratia KM, et al. Isoquinoline Kelch-like ECH-Associated Protein 1-Nuclear Factor (Erythroid-Derived 2)-like 2 (KEAP1-NRF2) Inhibitors with High Metabolic Stability. J Med Chem. 2020;63(12):6547-60.

7. Martinez VD, Vucic EA, Pikor LA, Thu KL, Hubaux R, Lam WL. Frequent concerted genetic mechanisms disrupt multiple components of the NRF2 inhibitor KEAP1/CUL3/RBX1 E3-ubiquitin ligase complex in thyroid cancer. Mol Cancer. 2013;12(1):124.

8. Clerici S, Boletta A. Role of the KEAP1-NRF2 Axis in Renal Cell Carcinoma. Cancers (Basel). 2020;12(11).

9. Tran KT, Pallesen JS, Solbak SMO, Narayanan D, Baig A, Zang J, et al. A Comparative Assessment Study of Known Small-Molecule Keap1-Nrf2 Protein-Protein Interaction Inhibitors: Chemical Synthesis, Binding Properties, and Cellular Activity. J Med Chem. 2019;62(17):8028-52.

10. Zhou H, Wang Y, You Q, Jiang Z. Recent progress in the development of small molecule Nrf2 activators: a patent review (2017-present). Expert Opin Ther Pat. 2020;30(3):209-25.

11. Mou Y, Wen S, Li YX, Gao XX, Zhang X, Jiang ZY. Recent progress in Keap1-Nrf2 protein-protein interaction inhibitors. Eur J Med Chem. 2020;202:112532.

**Supplemental Table S1**

Summary of the datasets collected from publicly available kidney tumor tissues versus normal tissue samples *

| **Tumor Abbreviation** | **KICH/chRCC** | **KIRC/ccRCC** | **KIRP/pRCC** |
| --- | --- | --- | --- |
| **Disease Type** | Kidney Chromophobe | Kidney Clear Cell Carcinoma | Kidney Papillary Cell Carcinoma |
| **TCGA samples** | 87 | 602 | 318 |
| **Tumor** | 64 | 531 | 286 |
| **Normal** | 23 | 71 | 32 |
| **Stage i** | 19 | 265 | 170 |
| **Stage ii** | 25 | 58 | 22 |
| **Stage iii** | 14 | 123 | 50 |
| **Stage iv** | 6 | 82 | 15 |
| **Stage Not reported** | 0 | 3 | 29 |

* The Table S1 is the summary of all the datasets that were downloaded from the GDC TCGA for the RNA-seq data for each of the three major types of RCCs (KICH/chRCC: 14 datasets; KIRC/ccRCC: 15 datasets; KIRP/pRCC: 15 datasets)

**Supplemental Table S2**

Somatic Mutation Analysis Summary of relevant genes *

| **Tumor Abbreviation** | **KICH/chRCC** | **KIRC/ccRCC** | **KIRP/pRCC** |
| --- | --- | --- | --- |
| **Disease Type** | Kidney Chromophobe | Kidney Clear Cell Carcinoma | Kidney Papillary Cell Carcinoma |
| **Samples** | 66 | 370 | 282 |
| **TP53 Mut** | 21/66 (31.82%) | 12/370 (3.24%) | 7/282 (2.48%) |
| **Kras Mut** | 0/66 | 2/370 (0.54%) | 5/282 (1.77%) |
| **BIRC5 Mut** | 0/66 | 0/370 | 0/282 |
| **NRF2 Mut** | 0/66 | 4/370 (1.08%) | 7/282 (2.48%) |
| **KEAP1 Mut** | 0/66 | 2/370 (0.54%) | 3/282 (1.06%) |
| **HIF1α Mut** | Not available | 5/370 (1.35%) | Not available |
| **HIF2α Mut** | Not available | 2/370 (0.54%) | Not available |
| **Mdm2 Mut** | 0/66 | 0/370 | 0/282 |
| **Mdm4/MdmX Mut** | 0/66 | 0/370 | 1/282 (0.35%) |
| **AKT1 Mut** | 0/66 | 1/370 (0.27%) | 0/282 |
| **AKT2 Mut** | 0/66 | 2/370 (0.54%) | 2/282 (0.71%) |
| **AKT3 Mut** | 0/66 | 1/370 (0.27%) | 0/282 |

*** Methods:** Somatic mutations data were downloaded from Broad Firehose (<http://firebrowse.org/>) and TCGA MC3 Project ^1^. Mutation Annotation Format (MAF) files were analyzed and visualized using R Bioconductor package, maftools ^2^.

Figures were produced using ggpubr and ggplot2 packages. Survival analysis was performed by utilizing the packages survival and survminer. Data analyses were performed using the R language (R version 4.0.3 *updated version*: [www.r-project.org](http://www.r-project.org)).

**References**

1 Ellrott K, Bailey MH, Saksena G, Covington KR, Kandoth C, Stewart C, et al. Scalable Open Science Approach for Mutation Calling of Tumor Exomes Using Multiple Genomic Pipelines. Cell Syst. 2018;6(3):271-81 e7.

2 Mayakonda A, Lin DC, Assenov Y, Plass C, Koeffler HP. Maftools: efficient and comprehensive analysis of somatic variants in cancer. Genome Res. 2018;28(11):1747-56.

**Supplemental Table S3**

Classification of KIRP/pRCC tumor and normal tissues into Type 1 pRCC and Type 2 pRCC

| **Kidney Papillary Cell Carcinoma (KIRP/pRCC)** | | | |
| --- | --- | --- | --- |
|  | **Normal** | **Tumor** | **Total** |
| **Type 1** | 4 | 76 | 80 |
| **Type 2** | 9 | 84 | 93 |
| **Type Not Reported** | 19 | 126 | 145 |
| **Total** | 32 | 286 | 318 |

| **Kidney Papillary Cell Carcinoma (KIRP/pRCC)** | | | | |
| --- | --- | --- | --- | --- |
| **Tumor** | **Type 1** | **Type 2** | **Type Not Reported** | **Total** |
| **Stage i** | 56 | 43 | 71 | 170 |
| **Stage ii** | 3 | 7 | 12 | 22 |
| **Stage iii** | 4 | 23 | 23 | 50 |
| **Stage iv** | 1 | 6 | 8 | 15 |
| **Stage Not reported** | 12 | 5 | 12 | 29 |
| **Total** | 76 | 84 | 126 | 286 |

**Supplemental Figure S1**

NRF2 expression in Type 1 pRCC and Type 2 pRCC tumor tissues versus in normal renal tissues: Boxplots of the NRF2 expression level across TCGA Type 1 or Type 2 pRCC tumor tissues (red) versus the normal renal tissues (blue) were presented (**A**). NRF2 expression among different stages of Type 1 pRCC tumor tissues versus the matched normal tissue (**B**) or versus all normal tissues (**C**) was box-plotted. NRF2 expression among different stages of Type 2 pRCC tumor tissues versus matched normal tissue was box-plotted (**D**). NRF2 expression was presented in the log2 (TPM+1) scale format. Data was presented as the mean ± standard deviation (SD). A t-test was used to evaluate the statistical significance of the NRF2 mRNA expression level in renal normal tissues versus either Type 1 or type 2 pRCC tumor tissues. One-way ANOVA was used to compare NRF2 expression among renal normal tissues versus different stages of Type 1 or Type 2 pRCC tumor tissues. The figure was performed using R version 4.0.3.

**Supplemental Figure S2**

Effects of NRF2 expression on Type 1 pRCC patient survival probability: Kaplan-Meier survival analyses of overall survival (OS) from TCGA-Type 1 (**A**) or Type 2 (**B**) pRCC cohorts were presented. Patients were grouped into the high NRF2 expression group versus the low NRF2 expression group based on the median NRF2 mRNA expression level in either Type 1 (**A**) or Type 2 (**B**) pRCC tumor tissues. Each p-value for the significance from high versus low NRF2 expression was calculated using the log-rank test. The figures were performed using R version 4.0.3.

**Supplemental Figure S3**

Mdm4/MdmX expression in RCC tumor tissues versus in normal tissues: Boxplots of the Mdm4MdmX expression level across TCGA-RCC subtypes in renal tumor (red) versus the associated normal renal tissues (blue) were presented (**A**). Mdm4MdmX expression among different stages of chRCC (**B**), ccRCC (**C**) and pRCC (**D**) versus normal renal tissues was box-plotted. Mdm4MdmX expression was presented in the log2 (TPM+1) scale format. Data was presented as the mean ± SD. A t-test was used to evaluate the statistical significance of the mRNA expression level in renal normal versus tumor tissues. One-way ANOVA was used to compare Mdm4MdmX expression among renal normal tissues versus different stages of RCC tumor tissues. The figure was performed using R version 4.0.3.

**Supplemental Figure S4**

Effects of Mdm4/MdmX expression on RCC patient survival probability: Kaplan-Meier survival analyses of OS from TCGA-RCC cohorts of ccRCC (**A**), chRCC (**B**) and pRCC(**C**) were presented. Patients were grouped into the high Mdm4/MdmX expression group versus the low Mdm4/MdmX expression group based on the median Mdm4/MdmX mRNA expression. Each p-value for the significance from high versus low Mdm4/MdmX expression was calculated using the log-rank test. The figures were performed using R version 4.0.3.

**Supplemental Figure S5**

AKT2 expression in RCC tumor tissues versus in normal tissues: Boxplots of the AKT2 expression level across TCGA-RCC subtypes in renal tumor (red) versus the associated normal renal tissues (blue) were presented (**A**). AKT2 expression among different stages of chRCC (**B**), ccRCC (**C**) and pRCC (**D**) versus normal renal tissues was box-plotted. AKT2 expression was presented in the log2 (TPM+1) scale format. Data was presented as the mean ± SD. A t-test was used to evaluate the statistical significance of the mRNA expression level in renal normal versus tumor tissues. One-way ANOVA was used to compare AKT2 expression among renal normal tissues versus different stages of RCC tumor tissues. The figure was performed using R version 4.0.3.

**Supplemental Figure S6**

Effects of AKT2 expression on RCC patient survival probability: Kaplan-Meier survival analyses of OS from TCGA-RCC cohorts of ccRCC (**A**), chRCC (**B**) and pRCC(**C**) were presented. Patients were grouped into the high AKT2 expression group versus the low AKT2 expression group based on the median AKT2 mRNA expression. Each p-value for the significance from high versus low AKT2 expression was calculated using the log-rank test. The figures were performed using R version 4.0.3.
